# Transcriptional dynamics of a conserved gene expression network associated with craniofacial divergence in Arctic charr

**DOI:** 10.1186/2041-9139-5-40

**Published:** 2014-11-03

**Authors:** Ehsan Pashay Ahi, Kalina Hristova Kapralova, Arnar Pálsson, Valerie Helene Maier, Jóhannes Gudbrandsson, Sigurdur S Snorrason, Zophonías O Jónsson, Sigrídur Rut Franzdóttir

**Affiliations:** Institute of Life and Environmental Sciences, University of Iceland, Sturlugata 7, 101 Reykjavik, Iceland; Biomedical Center, University of Iceland, Vatnsmýrarvegur 16, 101 Reykjavik, Iceland

**Keywords:** Arctic charr, Coexpression, Craniofacial development, Divergent evolution, Gene network, Morphogenesis, *Salvelinus alpinus*

## Abstract

**Background:**

Understanding the molecular basis of craniofacial variation can provide insights into key developmental mechanisms of adaptive changes and their role in trophic divergence and speciation. Arctic charr (*Salvelinus alpinus*) is a polymorphic fish species, and, in Lake Thingvallavatn in Iceland, four sympatric morphs have evolved distinct craniofacial structures. We conducted a gene expression study on candidates from a conserved gene coexpression network, focusing on the development of craniofacial elements in embryos of two contrasting Arctic charr morphotypes (benthic and limnetic).

**Results:**

Four Arctic charr morphs were studied: one limnetic and two benthic morphs from Lake Thingvallavatn and a limnetic reference aquaculture morph. The presence of morphological differences at developmental stages before the onset of feeding was verified by morphometric analysis. Following up on our previous findings that *Mmp2* and *Sparc* were differentially expressed between morphotypes, we identified a network of genes with conserved coexpression across diverse vertebrate species. A comparative expression study of candidates from this network in developing heads of the four Arctic charr morphs verified the coexpression relationship of these genes and revealed distinct transcriptional dynamics strongly correlated with contrasting craniofacial morphologies (benthic versus limnetic). A literature review and Gene Ontology analysis indicated that a significant proportion of the network genes play a role in extracellular matrix organization and skeletogenesis, and motif enrichment analysis of conserved noncoding regions of network candidates predicted a handful of transcription factors, including *Ap1* and *Ets2*, as potential regulators of the gene network. The expression of *Ets2* itself was also found to associate with network gene expression. Genes linked to glucocorticoid signalling were also studied, as both *Mmp2* and *Sparc* are responsive to this pathway. Among those, several transcriptional targets and upstream regulators showed differential expression between the contrasting morphotypes. Interestingly, although selected network genes showed overlapping expression patterns *in situ* and no morph differences, *Timp2* expression patterns differed between morphs.

**Conclusion:**

Our comparative study of transcriptional dynamics in divergent craniofacial morphologies of Arctic charr revealed a conserved network of coexpressed genes sharing functional roles in structural morphogenesis. We also implicate transcriptional regulators of the network as targets for future functional studies.

**Electronic supplementary material:**

The online version of this article (doi:10.1186/2041-9139-5-40) contains supplementary material, which is available to authorized users.

## Background

Unravelling the developmental and genetic basis of morphological and functional diversity is a fundamental step in understanding the evolutionary processes involved at both intra- and interspecific levels. The extensive diversity of teleost fish has been a fertile model system for studying such variation [1–5]. Closely related species or subspecies of fish are frequently distinguished by differences in the trophic apparatus, such as the shape and dynamics of jaws and pharyngeal elements [6]. We are interested in genes and mechanisms underlying the evolution of such differences. Explaining these mechanisms is a formidable challenge, however, as the trophic apparatus is a highly complex and integrated musculoskeletal system formed through interactions between derivatives of all three germ layers. Significant progress has already been made in studying the development of craniofacial elements in model species such as zebrafish, where genetic research has allowed detailed dissection of craniofacial development at the molecular level (reviewed in [7]). The accumulating knowledge about the structure and role of signalling pathways and gene expression differences in development is also becoming a significant tool for addressing questions at interspecific and phylogenetic levels. For example, such evidence was recently used in advancing a hypothesis about a developmental trade-off (constraints versus flexibility) influencing the radiation of the trophic apparatus in cichlids, a model species group for studying trophic radiation [8]. Recent advances in molecular techniques, such as whole-transcriptome sequencing (RNA-Seq) have opened new avenues for studying nonmodel species [9, 10]. In this respect, the polymorphic freshwater fish of northern postglacial lakes offer exciting opportunities for studying the developmental and genetic bases of rapid diversification in trophic structures and their relation to evolutionary forces [11].

Arctic charr (*Salvelinus alpinus*) is a highly suitable species for such studies. It shows extensive morphological variation throughout its geographic distribution and sports many cases of distinct polymorphisms which have evolved rapidly and repeatedly [12–14]. A striking example is seen in Lake Thingvallavatn in Iceland, where four resident morphs of Arctic charr are found: A large benthivorous (LB) morph, a small benthivorous (SB) morph, a planktivorous (PL) morph and a piscivorous (PI) morph [15]. In some ways, these morphs bear the hallmarks of separate subspecies, as they exhibit different ecological, behavioural and morphological characteristics [15–19] and show significant genetic divergence in neutral markers as well as in genes related to immunity [20, 21]. The two benthivorous morphs that appear to be derived have an overshot mouth, whereas the two limnetic morphs have a terminal mouth as well as shorter pectoral fins and a higher number of gill rakers [16]. Common garden experiments have shown that both variation in trophic morphology and feeding behaviour have a genetic basis, although maternal and environmental components also play a role [22–24]. Furthermore, these experiments show that some of the morphological differences arise early and are rooted in differential embryonic processes that could, for instance, evolve through heterochrony [22, 23]. Transcriptional heterochrony has been shown to contribute to craniofacial divergence in other fish species [25–27] and is likely to contribute to divergence of the Arctic charr morphs as well [23, 28].

Adaptations can arise through structural or regulatory changes in individual genes, several loci or coordinated deployment of coregulated genes [29, 30]. In developmental profiles, this would be reflected in differences in the timing, levels or patterns of gene expression. Previous studies on such differences between Arctic charr morphs have been focused on early embryonic development or posthatching stages in specific tissues [28, 31]. Data obtained from a preliminary transcriptome sequencing experiment indicated differential expression patterns during four developmental stages in two contrasting morphotypes of Arctic charr. Among these genes were *Mmp2* and *Sparc*, and, as a proof of principle, their differential expression in the developing head of benthic and limnetic morphotypes was verified by reverse transcription quantitative PCR (RT-qPCR) analysis [32]. Several studies suggest that both genes play an important role in craniofacial morphogenesis in vertebrates [33–37], and a positive correlation of *Mmp2* and *Sparc* expression levels has been reported [38–40], suggesting coregulation or synchronized biological function.

In view of this information, we decided to further investigate the expression dynamics and potential regulators of these genes. We wanted to find out whether *Mmp2* and *Sparc* might be part of a larger network of genes with correlated expression during craniofacial morphogenesis and test whether such a network would show differential expression in developing heads of contrasting Arctic charr morphotypes. To accomplish this goal, we identified genes with strong expressional correlation to *Mmp2* and *Sparc* in other species and selected those which also showed differential expression in developmental transcriptome profiles in contrasting Arctic charr morphotypes. Here we report that a network of functionally related genes shows coexpression in the developing head of Arctic charr embryos and is differentially expressed between benthic and limnetic morphotypes. The network genes share conserved binding motifs for a set of transcription factor (TFs), including *Ap1* and *Ets2*. Interestingly, *Ets2* itself is differentially expressed between the benthic and limnetic Arctic charr morphs during craniofacial development and shows strong expressional correlation with the network as well as spatiotemporal overlap in expression pattern.

## Methods

### Fish stocks, embryonic staging and sampling

Ripe parent fish from three of the Lake Thingvallavatn Arctic charr morphs—PL (small limnetic) morph, SB morph and LB morph—were sampled in 2010 during their respective spawning periods. For each morph, eggs from several females were pooled and fertilized using milt from several males. We also set up pooled crosses from a limnetic aquaculture stock (AC) from the Hólar College breeding programme. Eggs were reared at approximately 4°C to 5°C in hatching trays (EWOS, Bergen, Norway) under constant water flow and in complete darkness at Hólar College experimental facilities in Verið, Sauðárkrókur, Iceland. The water temperature was recorded twice daily, and the average was used to estimate the relative age of the embryos using tau-somite (τ_s_) units, defined as the time it takes for one somite pair to form at a given temperature [41].

### Morphometric analysis of the developing head

**Figure 1:**
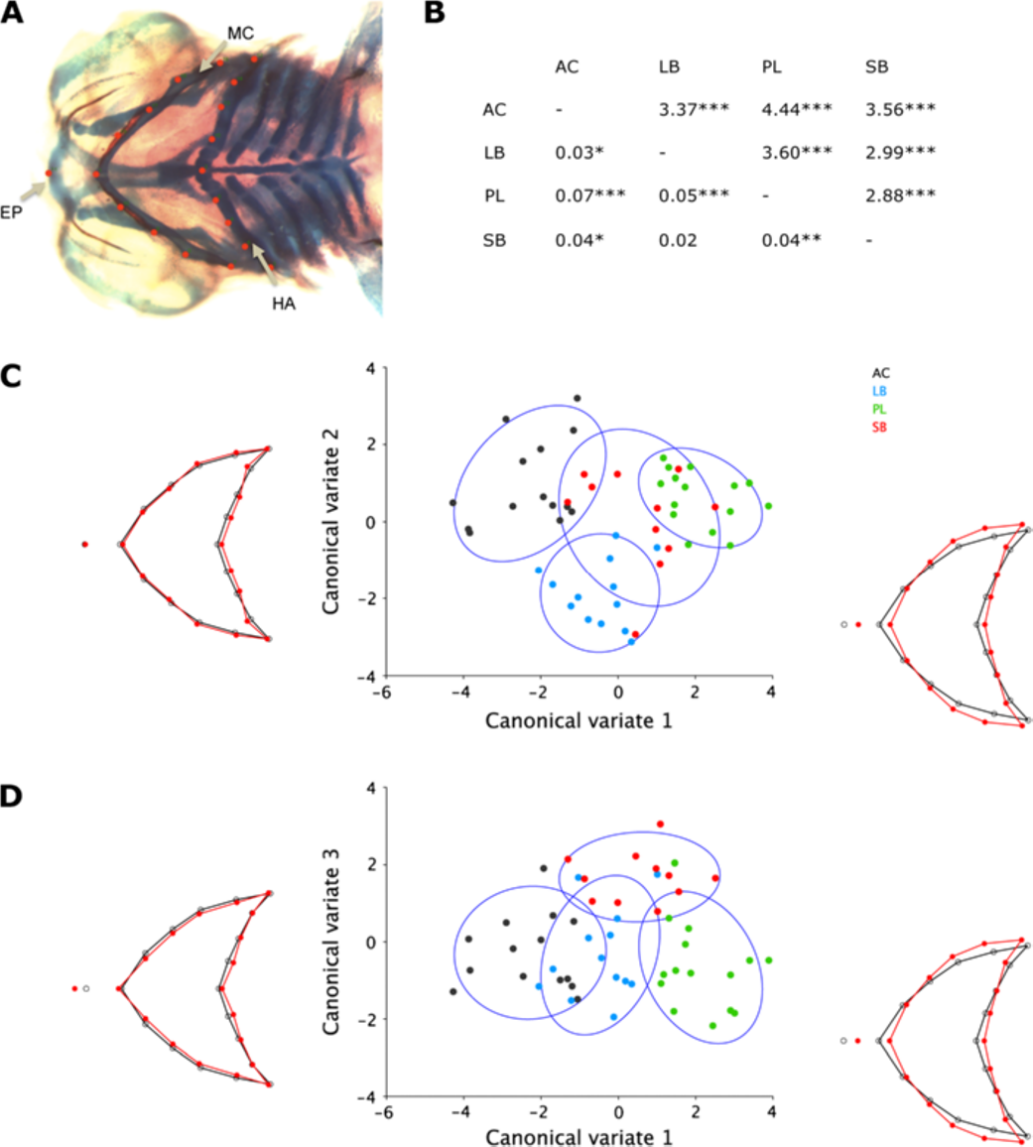
**Shape differences between morphs assessed with geometric morphometrics. (A)** Landmarks marking the anterior tip of the ethmoid plate (EP, leftmost landmark), lower jaw (MC, Meckel’s cartilage) and hyoid arch (HA). **(B)** Pairwise Mahalanobis (upper panel) and Procrustes distances (lower panel) between morphs and their significance obtained with 10.000 permutations. AC, Aquaculture charr from the Hólar breeding stock; LB and SB, Large and small benthivorous charr, respectively; PL, Planktivorous charr. ***P <0.001; **P <0.01; *P <0.05. **(C)** and **(D)** Scatterplots of the canonical variate (CV) analysis scores for four morphs of Arctic charr (AC, Black dots; LB, Blue dots; PL, Green dots; SB, Red dots). Wireframes depict shape changes associated with the two CVs shown in each graph (CV1 and CV2 in part (C) and CV1 and CV3 in part (D)). In the wireframes, the shape corresponding to the extreme negative CV score is shown in black, and the shape corresponding to the extreme positive CV score is shown in red. The scale factor is in units of Mahalanobis distance and is set to 6. Confidence ellipses are set to 90%.

For morphometric comparisons of PL, LB, SB and AC morphs, we selected newly hatched embryos (305 τ_s_). Samples were fixed in 4% paraformaldehyde. A total of 53 individuals (about 13 individuals per morph) were stained for cartilage (Alcian blue) and bone (Alizarin red) using a modified protocol for zebrafish [42]. The head of each individual was photographed ventrally under a dissecting microscope, and the same magnification (2.0×) was used for each photograph. Landmarks were selected to describe the shape of the lower jaw, its distance from the anterior tip of the ethmoid plate and the shape of the hyoid arch (Figure 1A) and digitised with tps.DIG2 [43]. Every individual was digitised three times, and the results from the repeated measurements were averaged in the final data set. The shape information for each specimen was extracted using a generalized Procrustes analysis in MorphoJ [44], where, after accounting for scale, position and orientation, all specimens were superimposed to a common coordinate system [45]. Only the symmetric component of shape variation [46] was used for subsequent statistical analysis. The centroid size (defined as the square root of the sum of the squared distances of all landmarks from their centroid) of each specimen was retained after the Procrustes fit and used as a measure of individual size. To remove the effect of allometry (morphological variation caused by differences in size), we used the residuals from the regression of shape on size for subsequent analysis. The differences between morphs were assessed using two statistical methods. First relative warp analyses were performed in tpsRelw [43], and the morph effect of each warp was tested with a generalized linear model in R [47]. Next, we generated two distances—(1) Mahalanobis distance (which measures the distances of separation between two groups scaled by the standard deviation in the respective directions) and (2) Procrustes distances (which measures the absolute amount of shape variation)—and assessed their statistical significance with 10.000 permutations. To visualize the differences between morphs, we used canonical variate analysis (CVA) in MorphoJ [44] and used differences between extremes to illustrate shape differences for CVA.

### Databases, gene coexpression and overrepresentation analysis

To gauge a potential network of coexpressed craniofacial genes, we searched COXPRESdb (http://coxpresdb.jp/) version 5.0 using orthologs of *Mmp2* and *Sparc* [48]. The 500 genes with tightest coexpression with both *Mmp2* and *Sparc* were retrieved for the three vertebrates with the largest available data sets (human, mouse and zebrafish) using the mutual rank (that is, the geometric mean of the correlation rank of gene A to gene B and of gene B to gene A). Genes with reliability scores less than three were discarded [48]. A total of 347 coexpressed genes were retained: 226 in humans, 176 in mice and 78 in zebrafish. In subsequent filtering steps, we selected genes showing differential expression between morphotypes in developmental transcriptome profiles of SB and AC morphs (see the Background section above; see also Gudbrandsson *et al.*, unpublished data) that also show craniofacial expression in zebrafish (according to the Zebrafish Model Organism Database (ZFIN) up to August 2013) [49].

The coexpression relationships between genes from the resulting list, along with selected glucocorticoid (GC) effectors (see reasoning below), were visualized across divergent vertebrate species using FunCoup version 2.0 [50]. FunCoup uses Bayesian statistics to estimate the probability of functional coupling between two genes, based on multiple data sets containing information on mRNA and protein interactions, and presents this as a probabilistic confidence value (pfc). We used FunCoup to map an interaction network based on mRNA coexpression consensus for several vertebrate species using a pfc cutoff above 0.5.

To characterize the coexpression module, we performed Gene Ontology (GO) overrepresentation analysis on top-ranked genes coexpressed with both *Mmp2* and *Sparc* in humans and mice (226 and 178 genes, respectively) using the Database for Annotation, Visualization and Integrated Discovery (DAVID) v6.7 [51]. The GO category used was ‘biological process’ at levels 3 to 5. Furthermore, to predict the potential regulators of the genes, TF enrichment analysis was conducted using the list of genes coexpressed with both *Mmp2* and *Sparc* in humans and mice, as well as the WEB-based GEne SeT AnaLysis Toolkit (WebGestalt) v2 [52].

### RNA isolation, cDNA synthesis and primer design for RT-qPCR

We studied embryos of four morphs, collected at seven time points spanning early craniofacial cartilage formation to a prehatching time point (155, 178, 200, 216, 238, 256 and 275 τ_s_), and, for simplicity, these time points will be referred to as stages 1, 2, 3, 4, 5, 6 and 7, respectively. Extraembryonic membranes were punctured, and the embryos were stored in RNA*later* solution (Ambion, Austin, TX, USA) at -20°C. For RNA extraction, embryos were dechorionated and decapitated in front of the pectoral fin under a light microscope (Leica S6 E; Leica Microsystems, Wetzlar, Germany). Two separate extractions were made for each morph and time point. For each replicate, six heads were placed in TRI Reagent (Sigma-Aldrich, St Louis, MO, USA) and homogenized with a disposable Kontes Pellet Pestle cordless motor tissue grinder (Kimble Chase, Rockwood, TN, USA). RNA was prepared according to the manufacturer’s instructions and dissolved in 50 μl of RNase-free water. To remove DNA contamination, the RNA was treated with DNase I (New England Biolabs, Ipswich, MA, USA). The quantity of the resulting RNA was measured using a NanoDrop ND-1000 UV/VIS spectrophotometer (NanoDrop Technologies, Wilmington, DE, USA). The quality of the RNA was evaluated by agarose gel electrophoresis by determining the integrity of the 18S and 28S RNA bands. cDNA was prepared from 1 μg of RNA in a total reaction volume of 20 μl using the High Capacity cDNA Reverse Transcription kit (Applied Biosystems, Foster City, CA, USA) according to the manufacturer’s protocol. The absence of genomic DNA was confirmed by preparing several samples without addition of reverse transcriptase. cDNA was diluted threefold in nuclease-free water for further use in qPCR experiments.

For qPCR primer design, we used a draft assembly of the Arctic charr transcriptome (Gudbrandsson *et al.*, unpublished data). We used the high conservation of exon–intron boundaries between orthologous genes and aligned charr contigs to the genomic sequences of zebrafish and salmon orthologs from the Ensembl database (http://useast.ensembl.org/Danio_rerio/Info/Index) and salmonids species database (http://salmondb.cmm.uchile.cl/) using the National Center for Biotechnology Information Spidey software (http://www.ncbi.nlm.nih.gov/spidey). Primers were designed using Primer Express 3.0 software (Applied Biosystems) and checked for self-annealing, heterodimers and hairpin structures by using OligoAnalyzer 3.1 (Integrated DNA Technologies, Coralville, IA, USA) (Additional file 1).

### Quantitative RT-PCR and analysis of expression data

RT-qPCR was performed in 96-well PCR plates on an 7500 Real-Time PCR System (Applied Biosystems) using 2× Fermentas Maxima SYBR Green qPCR Master Mix (Fisher Scientific, Pittsburgh, PA, USA) with a 10-μl final reaction volume. Each biological replicate was run in duplicate together with a no-template control in each run for each gene. RT-qPCR was performed as described previously [32]. Fluorescence signal baseline and threshold values were set manually using 7500 System SDS software (Applied Biosystems), generating a quantification of cycle (Cq) for each sample. Primer efficiency values (E) were calculated by using the LinRegPCR v11.0 programme (http://LinRegPCR.nl) [53] to analyse the fluorescence data from the exponential phase of PCR amplification for each primer pair (Additional file 1). The difference between Cq values (ΔCq) of the reference genes and the target genes was calculated for each gene *t* (target) as follows: ΔCq_target_ = Cq_target_ – Cq_reference_. The geometric mean of Cq values of two validated craniofacial reference genes, *If5a1* and *Actb*, was used for ΔCq calculations [32]. All samples were then normalized to the ΔCq value of a calibrator sample to obtain a ΔΔCq value (ΔCq_target_ – ΔCq_calibrator_). A biological replicate from AC at 155 (τ_s_) was chosen as the calibrator sample to calculate the differential mRNA expression of each target gene. Relative expression quantities (RQ) were calculated based on the expression level of the calibrator sample (E^-ΔΔCq^) [54]. The RQ values were transformed to logarithmic base 2 values (or fold differences, FD) [55] for statistical analysis. However, the nontransformed RQ values were preferred to visualize the relative expression differences. A two-way analysis of variance (ANOVA) test for the effects of morph, developmental stage (time) and morph × time interaction on expression of the candidate genes was implemented with a generalized linear model in R. Tukey’s honest significant difference (HSD) *post hoc* tests were used to contrast benthic and limnetic morphotypes. To assess expression similarity of the RT-qPCR-amplified genes, Pearson correlation coefficients (*r*) were calculated for all gene pairs using the data from all morphs and time points. R (http://www.r-project.org) was used for all statistical analyses [47].

### *In silico*analysis of transcriptional binding sites

Tests for the enrichment of motifs, aimed at uncovering potential transcriptional regulators, were conducted for 17 genes in the coexpression module showing expression differences between limnetic and benthic charr. The conserved noncoding regions of each gene (including sequences in promoter and 5′ untranslated regions conserved across fish species) were retrieved using the UCSC Genome Browser (http://genome.ucsc.edu) [56]. The conserved segments for each gene were concatenated and separated by strings of ‘N’ characters to avoid generating false binding sites. The motif enrichment analysis was conducted with two programs: SCOPE (http://genie.dartmouth.edu/scope) [57] and BioProspector [58]. SCOPE utilizes three algorithms—BEAM, PRISM and SPACER—to identify nondegenerate, degenerate and bipartite motifs, respectively. The results from all three algorithms were merged and ranked based on a significance value (Sig) in SCOPE using three fish species—*Danio rerio*, *Tetraodon nigroviridis* and *Oryzias latipes*—with annotated genome and available background sequences in SCOPE. We retained motifs that were present in noncoding regions near at least 12 (70%) of the 17 genes. We screened for potential TF binding sites using STAMP [59], with the motifs’ position weight matrices (PWMs) retrieved from the TRANSFAC database [60]. Similar analysis was carried out using the Gibbs sampling program BioProspector [58] by setting the motif width to 8 bp. BioProspector allows using the input sequences themselves as background, so we also ran a similar analysis on a fourth fish species, *Gasterosteus aculeatus*, which has high-quality genome sequences available for the conserved noncoding sequences of the genes under study.

### Whole-mount *in situ*hybridization

Whole-mount *in situ* hybridization was performed following a standard procedure adapted for Atlantic salmon [61]. Embryos from three time points representing the early craniofacial bone and cartilage formation were fixed in 4% (m/v) paraformaldehyde/phosphate-buffered saline, dehydrated in a graded methanol series and stored in 100% methanol. Primers designed for cDNA of selected Arctic charr genes (*Ctsk*, *Ets2*, *Mmp2*, *Ogn*, *Sfrp1*, *Sparc* and *Timp2*) generated PCR products of around 400 to 700 bp (Additional file 1), which were cloned into a pCR4-TOPO vector (Invitrogen, Carlsbad, CA, USA) and transcribed to antisense/sense digoxigenin (DIG)-labelled cRNA probes with T3/T7 RNA polymerases (Roche Diagnostics, Basel, Switzerland). Depending on the gene, four to six embryos from each tested morph were used for *in situ* hybridization at each time point. After rehydration and dechorination, embryos were treated with 20 to 40 μg/ml proteinase K (New England Biolabs) for 20 to 60 minutes, depending on the developmental stage. The hybridization was performed with 1 μg/ml DIG-labelled RNA probes at 70°C for 12 hours. The hybridized embryos were incubated with alkaline phosphatase-conjugated anti-DIG antibody (Roche Diagnostics) at 4°C overnight, and the hybridization signals were visualized using nitro blue tetrazolium chloride/5-bromo-4-chloro-3-indolyl-phosphate, toluidine salt (NBT/BCIP; Roche Diagnostics). The specificity of antisense probes was also verified by running control experiments with sense probes. Samples were imaged on a Leica MZ10 F binocular microscope (Leica Microsystems).

## Results

Our primary goal in this study was to analyse differences in gene expression which might contribute to the benthic–limnetic craniofacial divergence in Arctic charr. We first set out to test whether morphological differences in craniofacial elements, particularly in the feeding apparatus, are present from an early developmental stage and are not the result of plasticity induced by different feeding behaviours. Embryos reared under identical conditions were sampled upon hatching, long before first feeding, and geometric morphometrics were performed to quantify differences in craniofacial morphology (Figure 1). Our results show differences between Arctic charr morphs in both jaw and hyoid arch morphology. The first three warps (essentially principal components of shape) accounted for 71.56% of the variance. W1 (warp 1), which accounted for the largest amount of variance (44.70%) had a highly significant (*P* <0.001 by ANOVA) morph effect and W2 (16.59%) also differed between morphs (*P* <0.05), whereas W3 (10.27%) did not. Pairwise comparisons using Mahalanobis distances showed significant differences between morphs (Figure 1B, upper panel). Although significant for most comparisons (the Procrustes distances between LB and SB were not statistically significant), the shape changes between morph pairs measured by Procrustes distances were subtle (Figure 1B, lower panel). The craniofacial shape differences between morphs were further characterized with CVA. Shape changes associated with CV1 (58.5%) included changes in both the lower jaw and the hyoid arch (Figure 1C). The lower jaw is more pointed and not as wide laterally in AC as in the Thingvallavatn morphs. CV2 (25.5%) showed more subtle changes in the shape of the lower jaw and hyoid arch (Figure 1C). CV3 (16%) showed subtle changes in the shape of the hyoid arch and the distance to the ethmoid plate (Figure 1D). SB showed signs of a more subterminal mouth at this early stage. Taken together, these data confirm that distinct morphological differences are present upon hatching, well before the juveniles start active foraging and become exposed to different environmental factors that can induce differential plastic responses that affect morphology.

### A conserved gene expression network with a potential link to glucocorticoid signalling

**Figure 2:**
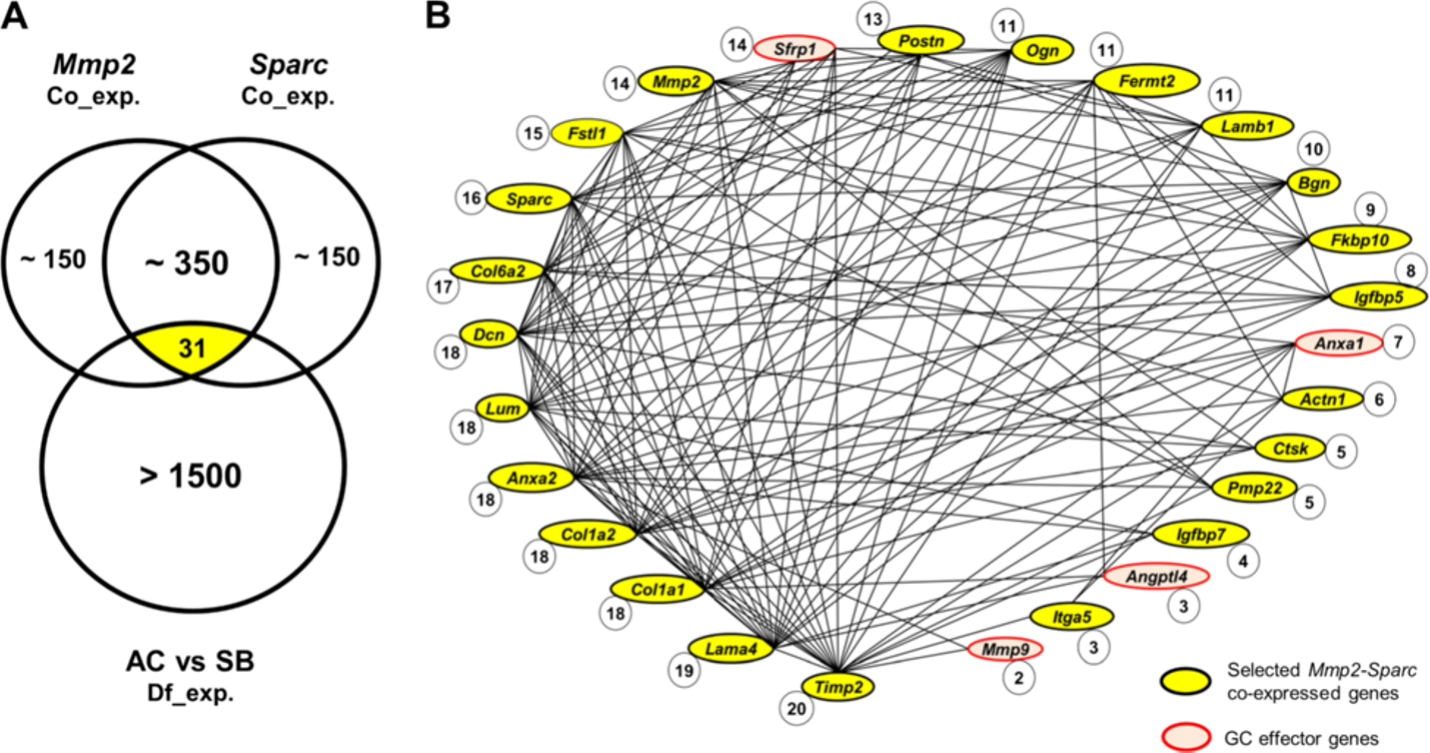
**Selection of genes coexpressed with** ***Mmp2*** **and** ***Sparc*** **in three vertebrate species. (A)** A Venn diagram illustrating the overlap between *Mmp2-Sparc* coexpressed genes and genes showing expression differences between Arctic charr morphs in a transcriptome profile. Of the 350 genes coexpressed with both *Mmp2* and *Sparc*, 31 genes (yellow) are differentially expressed between the aquaculture (AC) and small benthivorous (SB) morphs. **(B)** Of the 31 differentially expressed genes, 22 were selected on the basis of craniofacial expression in zebrafish (yellow circles), and their coexpression relationship in three vertebrate species (humans, mice and zebrafish) was depicted using FunCoup. Genes associated with glucocorticoid (GC) signalling were included in the analysis, and four of those (red circles) showed significant connection to the network. The number of connections within the network is shown beside each gene. A cutoff probabilistic confidence value of 0.5 was applied to remove the weak interactions.

As outlined in the Background section above, we previously showed that two matrix remodelling factors, *Mmp2* and *Sparc*, are differentially expressed between benthic and limentic Arctic charr morphotypes during the morphogenesis of craniofacial elements [32]. The expression of *Mmp2* and *Sparc* was further analysed in dense series of samples, spanning early craniofacial chondrogenesis up to a prehatching stage (Additional file 2). The expression dynamics of these two genes were highly similar, and clear differences between benthic and limnetic morphs were observed. We therefore decided to test the hypothesis that *Mmp2* and *Sparc* are a part of a larger expression module, or network of genes, differentially regulated between Arctic charr morphs during this stage of development. We asked which genes have conserved coexpression relationships with *Mmp2* and *Sparc* across divergent vertebrate species. We used COXPRESdb [48] to retrieve the genes showing the strongest coexpression with both *Mmp2* and *Sparc* in three vertebrate species (human, mouse and zebrafish). Of 347 genes coexpressed with both genes in at least one of these species, 150 were expressed during Arctic charr embryonic development, and 31 of those genes were among the genes suggested to be differentially expressed between benthic and limnetic morphotypes, based on preliminary transcriptome profiles from the SB and AC morphs (Figure 2A and Additional file 3; Gudbrandsson *et al.* unpublished data). Twenty-two of these genes were selected for further analysis, based on craniofacial expression pattern in zebrafish (according to the ZFIN database [49]). Figure 2B depicts the reported expression relationship between these genes, based on FunCoup analysis [50]. To characterize this coexpression module, we performed GO overrepresentation analysis on the top-ranking coexpressed genes in humans and mice (Additional file 3). The coexpression module is enriched for genes involved in extracellular matrix (ECM) organization, bone development and ossification. TF enrichment analysis on the same genes predicted several TFs as potential upstream regulators of the genes in both humans and mice (Additional file 3).

Potential regulators of the developmental differences between Arctic charr morphotypes are of particular interest. Three observations led us to include GC signalling in the analysis. First, both *Mmp2* and *Sparc* are known to be directly responsive to GC [35, 62]. Second, several of the 31 genes coexpressed with both *Mmp2* and *Sparc*, which also showed benthic–limnetic differential expression, are known to be responsive to GC (for example, *Anxa2*, *Ctsk*, *Dcn*, *Igfbp7*, *Itga5*, *Ogn* and *Pmp22* [63–68]). Third, GC signalling itself has profound effects on craniofacial morphogenesis in different vertebrate species [35, 69, 70]. Thus, we decided to examine whether other target genes and upstream effectors of GC signalling might fit to the network and show similar differences between morphs. We selected eight transcriptional targets: *Mmp9*, *Mif*, *Ocln* and *Sfrp1*, all of which show expression in the head mesenchyme and/or pharyngeal arch skeleton during zebrafish development; and *Anxa1*, *Angptl4*, *Nr4a1* and *Sgk1*, which have less restricted expression patterns, according to the ZFIN database [35, 49, 71–77]. The eight GC upstream effectors include the GC receptor *Nr3c1*, two enzymes predicted to control GC levels (*Hsd11b1l* and *Hsd11b2*) and a few known modulators of GC activity, that is, the TFs *P300*, *Cebpa*, *Ets1* and *Ets2*, and *Star*, an upstream component of steroidogenesis [78–84]. In addition, the TF *Srebp1*, which regulates the expression of *Star* [85] and showed differential expression between morphs in the transcriptome profiles, was selected for further analysis.

FunCoup was used to address the potential relationship between the previously selected coexpression network genes and these GC-related genes using the same cutoff value as before (pfc >0.5). Four of the GC target genes showed conserved coexpression with the network (*Angptl4*, *Anxa1*, *Mmp9* and *Sfrp1*) (Figure 2B).

### Proposed network genes show differential expression in the head of benthic and limnetic morphs

**Figure 3:**
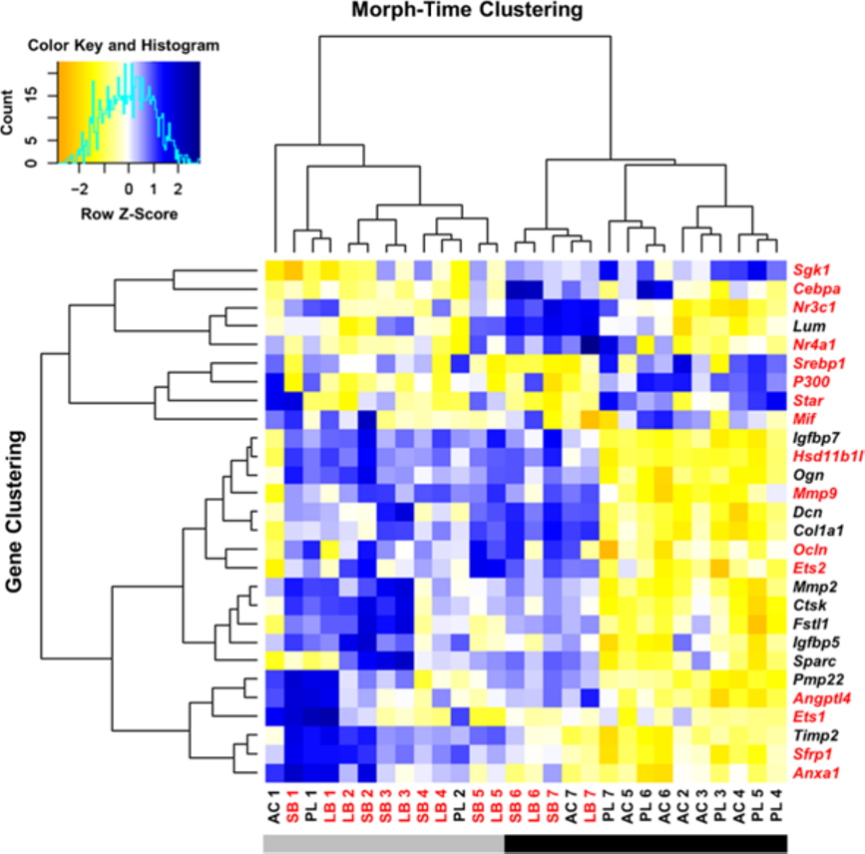
**Distinct morph- and time-dependent gene expression in the developing head of Arctic charr.** Heat map showing the relative expression of candidate network genes (black) and 16 genes associated with glucocorticoid (GC) signalling (red) in two limnetic morphs (black) and two benthic morphs (red) at seven developmental stages. Hierarchical clustering was performed on genes (vertical axis) and on samples (morph and timepoint, horizontal axis). Blue represents higher expression and yellow lower expression relative to the average levels across all samples. The bar below the figure underlines the two major branches of sample clustering.

To characterize the expression of the conserved coexpression network in the developing head of Arctic charr, we profiled the relative expression of *Mmp2*, *Sparc* and ten other members of the network in four morphs at seven consecutive time points (spanning early craniofacial bone and cartilage formation to the prehatching stage). qPCR was performed on samples extracted from dissected heads of SB, LB, PL and AC embryos, and the relative expression of the genes was normalized to the expression of two stable craniofacial reference genes, *Actb* and *If5a1* [32] (Additional file 2). The expression of all 12 genes differed significantly, both between two or more morphs and over time (by overall ANOVA and Tukey’s HSD *post hoc* tests) (Additional files 2 and 4). Interestingly, all of the genes showed consistently higher expression in the benthic morphs (LB and SB). The magnitude of the expression differences varied, and, for instance, *Lum* and *Igfbp5* displayed only slight but significantly higher expression in benthic heads, whereas *Timp2* had the largest expression difference (3.8-fold). The expression over time also varied by morph for all genes (morph × time effect in Additional file 4). Furthermore, an ANOVA on morphotypes confirmed significant expression differences between benthic (LB and SB) and limnetic (PL and AC) morphotypes for all 12 genes (Additional file 5). All of the proposed network genes except *Lum* clustered (Figure 3, black gene symbols). Nine of the genes clustered tightly, whereas *Pmp22* and *Timp2* were on a distinct branch together with genes associated with GC signalling (Figure 3, red gene symbols). We also clustered on samples (morph and time point) and observed significant associations of morphotypes on the two major branches (there were 4 benthic stages among 11 limnetic on one branch, and 10 benthic stages with 3 limentic on the other branch; *χ*^2^ test, *df* = 1, *P* = 0.023). A closer look at the clusters revealed that gene expression in the PL and benthic morphs was more similar at the first two time points (stages 1 and 2), whereas AC showed expression similar to benthic morphs at the last time point (stage 7) (Figure 3). Taken together, these results show significantly higher expression of members of the coexpression network in developing heads of benthic morphs.

### Genes related to glucocorticoid signalling show transcriptional correlation with the network genes

To test whether the GC signalling pathway is involved in the observed differences in head development among Arctic charr morphs, we studied the expression of the 16 selected effectors and targets of the GC pathway. The expression of *Hsd11b2* was below the detection limit of qPCR in all samples and was therefore not studied further. All of the eight downstream targets of the GC signalling pathway showed differential expression between the morphs at one or more time points (Additional files 4 and 6). Five of those—*Angptl4*, *Anxa1*, *Mmp9*, *Ocln* and *Sfrp1*—had higher expression in benthic morphs, similar to the twelve coexpressed genes described above (see Additional files 5 and 6). This is consistent with the coexpression data from other vertebrates, which place these genes (except *Ocln*) in the conserved coexpression network (Figure 2B). *Sgk1* was the only gene with significantly lower expression in benthic morphs over several time points (*P* <0.01) (Additional files 5 and 6).

Two of the eight selected effectors of the GC pathway (the enzyme *Hsd11b1l* and the TF *Ets2*) showed consistently higher expression in developing heads of the benthic morphs (Additional files 5 and 7). Both genes also clustered tightly with the members of the conserved coexpression network (Figure 3). The expression of the GC receptor (*Nr3c1*) differed slightly but significantly between the limnetic and benthic morphs, but the receptor did not cluster with genes from the coexpression network. As shown in Figure 3, most of the GC-related genes are arranged on a separate branch in the hierarchical clustering. In summary, several targets of the GC pathway showed tight clustering with genes of the proposed coexpression network. Expression of the GC receptor *Nr3c1* did not overlap completely with differences in network gene expression between morphs, but two effectors of the pathway—the activator *Hsd11b1l*, and *Ets2*, which can function as a coactivator for *Nr3c1* transcriptional activity [79, 83]—show expression dynamics highly similar to those of the network genes.

### Expression correlation analysis confirms a gene expression module differentially expressed between morphotypes

**Figure 4:**
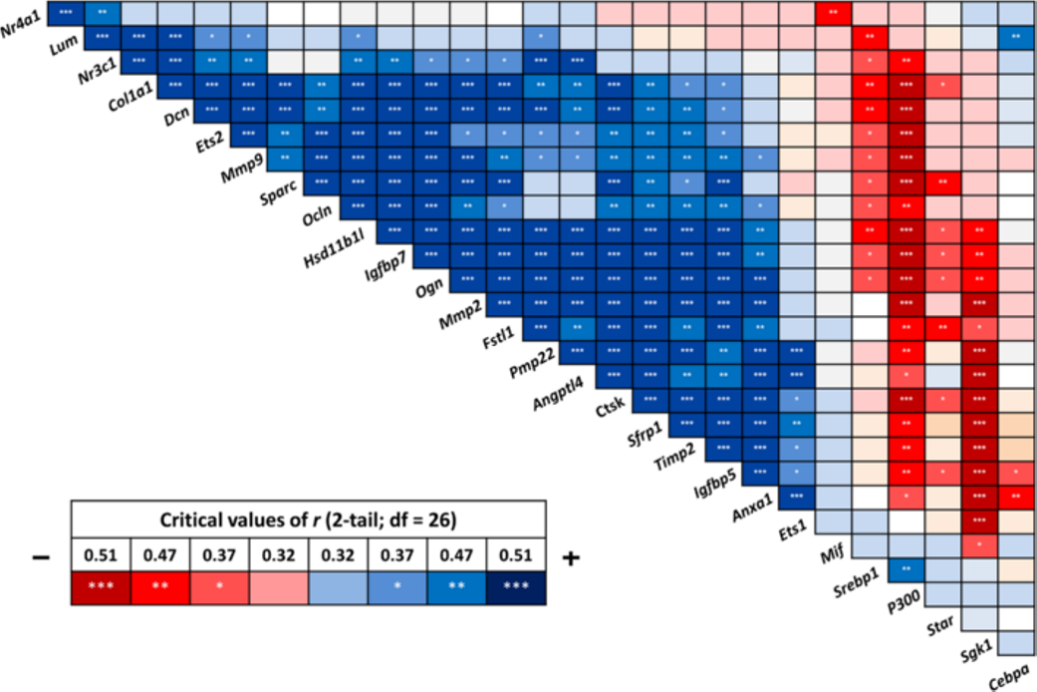
**Correlation analysis reveals significant positive or negative coexpression of the candidate genes.** Pearson correlation coefficient (*r*) was used to assess the pairwise expression similarity between the candidate genes during craniofacial development. Blue represents positive and red represents negative expression correlation. A scale showing critical values of *r* is depicted with corresponding colours. *P <0.05; **P <0.01; ***P <0.001.

To confirm that the genes in the network identified above in other species are indeed coexpressed in Arctic charr, we calculated the Pearson correlations of expression levels of the entire data set (all 28 genes) over all morphs and time points (Figure 4). A strong correlation was seen between the genes of the proposed network (blue in Figure 4), whereas most of the GC effectors with no predicted relation to the network (according to FunCoup analysis) showed either little or negative correlation with the network genes. However, three genes from the GC set, for which less data are available in the coexpression databases (*Ets2*, *Hsd11b1l* and *Ocln*), had strong positive correlation with almost all of the genes in the network. Interestingly, a few genes showed significant negative correlation with the network genes (whereby the increased expression of one gene is associated with decreased expression of another), most notably *P300* and *Sgk1. Lum*, which was originally predicted to show coexpression with *Mmp2* and *Sparc*, does not appear to form a part of this coexpression module in developing Arctic charr heads. To conclude, the analysis confirms the existence of a coexpression network conserved between vertebrates (Figure 2B), which appears to be differentially regulated during the development of morphologically distinct groups of Arctic charr.

### Bioinformatics analysis reveals potential upstream regulators of the network

The data suggest that a conserved coexpression module, including bone formation genes and GC signalling components, may be associated with morphological differences in the heads of benthic and limnetic Arctic charr morphs. The tight coexpression of those 17 genes differentially expressed in benthic versus limnetic charr points to the involvement of upstream regulators. In pursuit of TFs that may regulate this expression module, we screened for overrepresented motifs in conserved noncoding regions of these 17 genes. For each gene, sequences from three fish species (*D. rerio*, *T. nigroviridis* and *O. latipes*) were retrieved, and SCOPE was used to screen for enriched motifs. From a list of 40 to 60 significantly enriched motifs in each fish species (Additional file 8), we retained only those present in noncoding regions of at least 12 (70%) of 17 genes. The 12 to 23 motifs per species were queried against known TF PWMs, and, by retaining only those present in all 3 species, a total of 9 potential TFs remained (Table 1). A complementary analysis with BioProspector, in which a fourth fish species, *Gasterosteus aculeatus*, could be added, yielded predicted binding sites for only four TFs: *Atf2*, *Ets1*, *Ets2* and *Tel2* (Additional file 8). In summary, the results of the motif sequence enrichment analysis suggest several TFs which may regulate most of the 17 coexpressed genes studied (Table 1). It is particularly interesting to see *Ets2* among those potential regulators, as this TF is more highly expressed in benthic Arctic charr morphs and shows tight coexpression with the network genes.

**Table 1:** **Transcription factors binding sites identified in noncoding regions of coexpressed genes in three fish species**

Transcription factor	Transcription factor family	Motifs (coverage) and species	Matrix ID	***D. rerio***	***O. latipes***	***T. nigroviridis***
				E-value	E-value	E-value
*Ap1*	Basic region leucine zipper		M00925	3.40E-05	2.98E-03	2.98E-03
		AACTCA (82%)_D.re	M00174	1.00E-04	–	–
		CCTCA (94%)_O.la	M00199	1.40E-04	–	–
		CCTCA (88%)_T.ni	M00924	1.70E-04	–	–
*Ets2*	E-26 transformation specific	CTTCA (94%)_D.re	M00340	–	1.40E-08	9.18E-05
		ACAGGAA (75%);				
		CTTCA (100%)_O.la	M00771	1.00E-04	7.38E-06	–
		ACAGG (94%)_T.ni				
*Lmaf*	Basic region leucine zipper	GTTGAC (71%)_D.re	M01139	5.80E-04	1.91E-03	2.06E-03
		AGCAA (88%); CCAGC (88%)_O.la				
		CCTGA (88%)_T.ni				
*Lyf1*	Zinc finger DNA binding	CTCTCC (77%)_D.re	M00141	3.20E-04	5.27E-06	5.08E-05
		CTCCC (81%)_O.la				
		GGAGA (100%)_T.ni				
*Maz*		RGGKANNGA (77%); CTCTCC (77%); CTCHNTCC (71%)_D.re	M00649	2.50E-05	8.92E-07	1.18E-03
		CCTCA (94%); CTCCC (81%)_O.la				
		CAGGG (88%); GGAGA (100%); CCTCA (88%); AAGGG (88%)_T.ni				
*Nfkb*	Rel homology domain		M00051	–	8.21E-03	–
		CTCTCC (77%)_D.re	M00052	–	–	4.49E-06
		DGRADB (100%)_O.la	M00054	–	–	7.14E-05
		GGAAA (88%)_T.ni	M00194	3.80E-03	–	3.87E-05
			M00208	3.30E-04	–	3.49E-05
*Sf1*	Nuclear hormone receptor	CAAGC (100%)_D.re	M00727	1.00E-03	2.15E-06	1.00E-03
		AGGTC (100%)_O.lat	M01132	2.02E-03	4.56E-06	1.32E-03
		CATGG (77%); AAGGG (88%); AGGCC (71%)_T.ni				
*Smad3*	Mothers against decapentaplegic	CAGAC (94%); TCTGG (88%); TCTGT (88%)_D.re	M00701	2.60E-06	6.00E-06	2.32E-03
		TCTGT (100%)_O.la				
		CAGAG (94%); CAGAA_T.ni				
*Smad4*		CAGAC (94%)_D.re	M00733	1.38E-03	8.52E-04	8.52E-04
		GCAGC (81%)_O.la				
		GCAGC (88%)_T.ni				

Conserved noncoding regions of 17 genes, with differential expression between morphs and positive expression correlation were analysed in three fish species (*D. rerio*, *T. nigroviridis* and *O. latipes*). Motifs present in at least 12 of 17 genes (70% coverage) were identified by SCOPE and run against position weight matrices in the TRANSFAC database to identify known transcription factor binding sites.

### Selected network genes show similar spatiotemporal expression patterns

**Figure 5:**
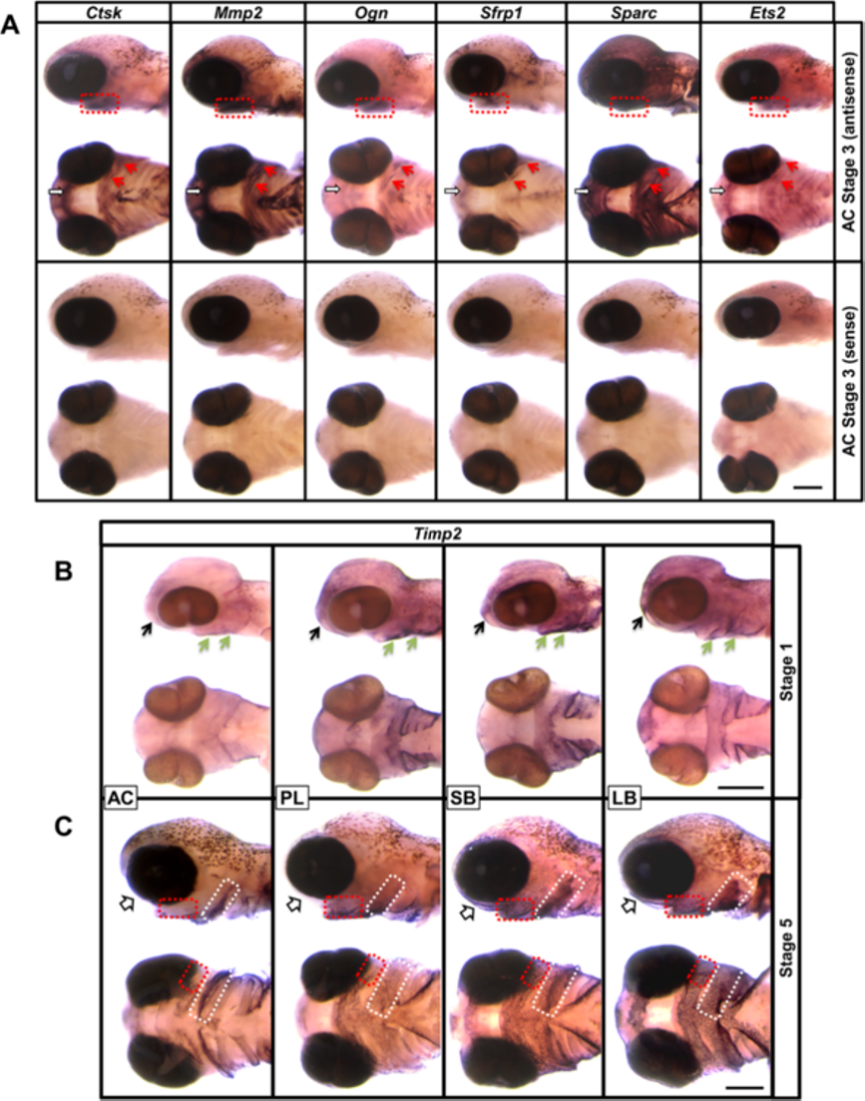
**Craniofacial expression pattern of selected members of the coexpression network. (A)** Whole-mount *in situ* hybridization for *Ctsk*, *Mmp2*, *Ogn*, *Sfrp1* and *Sparc* at stage 3 in the aquaculture (AC) morph, ventral and lateral views. Expression of all genes can be seen anterior to and surrounding the mouth (white arrows), as well as in the pharyngeal arches. Overlapping expression is highly pronounced in the perichondrial region of the lower jaw (red arrows and dashed squares). **(B)** Comparison of *Timp2* expression between the four morphs at stage 1. An overall weaker staining is obtained for *Timp2* in AC compared with the other morphs. Pronounced expression of *Timp2* can be seen in the frontonasal region (black arrows) and at the pharyngeal arches (green arrows). **(C)** At stage 5, both AC and the planktivorous (PL) morph show weaker expression of *Timp2* in an area anterior to the mouth (white arrows). A clear difference between the benthic and limnetic morphs can also be seen in the expression pattern and levels of *Timp2* in the pharyngeal arch region (red and white dashed squares highlight some of the differences). LB, Large benthivorous morph; SB, Small benthivorous morph. Scale bar = 1 mM.

After demonstrating quantitative differences in gene expression between morphs, we set out to analyse spatial and temporal expression patterns of the coexpression network genes in the developing head of Arctic charr. We wanted to know whether the network genes were expressed in the same tissues within the head and whether any detectable expression pattern differences would be seen between morphs. Five differentially expressed network genes (*Mmp2*, *Sparc*, *Ctsk*, *Ogn* and *Sfrp1*) were initially selected for whole-mount *in situ* hybridization studies to investigate their expression patterns, using AC as a reference morph. The expression pattern of the genes was profiled at the earliest, intermediate and latest time points under study (that is, stages 1, 3 and 7) (Figure 5A and Additional file 9). All five genes showed craniofacial expression, which was particularly pronounced in anterior and ventral facial elements and pharyngeal arches. Overlap in expression pattern was most evident in the facial area anterior to and surrounding the mouth, as well as in the lower jaw. Interestingly, at stage 3, all five genes showed a pronounced perichondrial pattern in the lower jaw (red arrows in Figure 5A).

As *Ets2* was identified as a potential regulator of the network which also showed expression dynamics that correlated with the other network genes under study, we were interested in analysing its expression *in situ*. Indeed, we found *Ets2* to be expressed in a pattern highly similar to that of the other genes (Figure 5A), which further supports our suggestion that *Ets2* may be a key component in driving expression of the network genes.

No major spatial expression pattern differences were observed between the morphs (data not shown), indicating that the differential expression observed in qPCR experiments was caused mainly by differences in expression levels within tissues and not due to spatiotemporal differences in expression patterns. However, when the expression of *Timp2*, the factor that showed the strongest expression differences between benthic and limnetic morphs by qPCR (Additional file 2) was analysed *in situ*, we did observe significant differences in expression patterns between AC and the other morphs (Figures 5B and 5C). *Timp2* expression was most pronounced on the ventral surface of the head. Consistent with the qPCR results, *Timp2* had the lowest expression in AC at stage 1, whereas the expression was weaker in AC and PL at stage 5 than in the benthic morphs. It should be noted that at stage 5 the pattern of *Timp2* expression also differed between AC and the other three morphs, with more restricted expression in posterior pharyngeal arch regions and less expression in the first pharyngeal arch and the area surrounding the mouth. Although the expression pattern of *Timp2* in PL was more similar to the benthic morphs, it was reduced in an area anterior to the mouth and certain regions around the pharyngeal arches (see Figures 5B and 5C). In summary, five selected network genes (*Mmp2*, *Sparc*, *Ctsk*, *Ogn* and *Sfrp1*) and *Ets2*, a potential upstream regulator, showed similar overlapping craniofacial expression in anterior and ventral facial elements and pharyngeal arches in all four charr morphs. Only *Timp2* showed spatiotemporal differences between the morphs.

## Discussion

In this study, we have taken steps towards uncovering molecular mechanisms associated with the rapid and extensive phenotypic divergence of Arctic charr in Icelandic lakes. Following up on our previous finding that two ECM-associated factors related to bone development, *Mmp2* and *Sparc*, are expressed at higher levels in benthic than in limnetic morphs [32], we asked whether they might be part of a larger set of developmental genes under common regulation, which might have a function in craniofacial development and thus contribute to the trophic differences between the benthic and limnetic morphotypes. We mined coexpression databases for genes showing coexpression with both *Mmp2* and *Sparc* in different vertebrate species. Coexpressed genes showing craniofacial expression in zebrafish and differential expression in transcriptional profiles of two contrasting Arctic charr morphs (Gudbrandsson *et al.*, unpublished data) were then selected for further study. This allowed the identification of a conserved network of genes which is differentially expressed between benthic and limnetic Arctic charr morphs, as well as the identification of potential upstream transcriptional regulators.

### Differential expression of network genes in the developing heads of contrasting Arctic charr morphs

The fact that all the genes from the initial list of coexpression candidates that we examined in this study showed correlated expression dynamics and significantly higher expression in benthic than limnetic morphs supports the idea that a coexpression network including *Mmp2* and *Sparc* is differentially regulated in the morphotypes. Distinct coexpression modules have been found to correlate with specific developmental stages or structures, such as in the zebrafish blastula and segmentation [86]. Although some coexpression modules are highly conserved, drift or natural selection can affect key regulators and bring about evolutionary changes [87, 88]. For instance, analyses of lake whitefish transcriptomes from adult tissues found associations between bone morphogenetic protein and calcium signalling coexpression modules, as well as the evolution of differences in the trophic apparatus and behaviour [30].

Despite the generally tight expressional correlation between the network genes, a few of the initially predicted network genes showed only partial pairwise correlation with the rest of the network. *Lum* is one of these genes (Figures 3 and 4). In spite of tight association with the expression of other genes of the network in other species, *Lum* expression dynamics correlated with only a few of them in developing Arctic charr heads. As with many of the other network genes, *Lum* is an ECM component and affects collagen fibril formation [89]. In Atlantic salmon, *Lum* is expressed at sites of endochondral and intramembranous ossification [90] and is a component of developing bone tissue in other vertebrate species [89], similarly to many of the other network genes. However, *Lum* is also a major proteoglycan in the cornea and sclera [91, 92], where it is likely to be independently regulated. This could mask more subtle expression dynamics and differences in other tissues of the developing head.

### Potential involvement of glucocorticoids in network gene expression

The association of multiple GC transcription targets with the network led us to ask whether GC activity might be different between benthic and limnetic morphs. This is, of course, a difficult question to approach at the transcriptional level, but higher expression of an activating factor, together with higher expression of multiple target genes, might imply differential GC activity in the morphs. Sixteen genes with different association with GC signalling (steoroidogenic enzymes, the GC receptor, several transcriptional cofactors and well-established GC target genes) were selected for further study. We found that five of the eight additional GC transcriptional targets were differentially expressed between benthic and limnetic morphs and showed strong expressional correlation with the network genes (see Figure 3). Furthermore, the GC effectors *Hsd11b1l* and *Ets2* showed a similar pattern.

During cranial skeletal development of the mouse, GC signalling has been suggested to regulate Wnt signalling in early osteoblastogenesis [69]. Wnt signalling is a pivotal pathway in controlling cranial bone formation [93] and has recently been strongly associated with the evolution of distinct craniofacial phenotypes in Lake Malawi cichlids [8]. The regulation of Wnt signalling by GCs might be conducted through an effect on the expression of the Wnt antagonist *Sfrp1* [77, 94]. In zebrafish, supraphysiological GC treatment during development can lead to craniofacial abnormalities [35]. Moreover, the craniofacial abnormalities might be exerted through the other GC-responsive genes, such as *Anxa1*, *Ctsk* and *Dcn*, which have known effects on craniofacial formation [95–97]. GCs activate transcription through the cognate receptor *Nr3c1* (GR) [98]. In addition to the action of GC through the receptor, the enzymes *Hsd11b1* and *Hsd11b2* modulate GC metabolism within the cell at the prereceptor level by catalysing the interconversion of hormonally active cortisol and inactive cortisone [99]. In fish, an ancestral enzyme gene named *11β-hsd3* (*Hsd11b1l*) is present instead of *Hsd11b1* [79]. Our findings show that *Hsd11b1l* expression correlates well with the network genes and that *Nr3c1* is expressed at higher levels in benthic morphs at certain timepoints. It is therefore possible that higher levels of GCs are present in the benthic morphs as well as a more robust response (higher GR levels).

Together, the data imply a possible interplay of GC signalling with the coexpression network which would be either direct, by driving network gene expression, or indirect, as a consequence of affecting effectors of GC activity. Analysis of conserved noncoding regions of the genes under study did not show significant enrichment for motifs matching GC-responsive elements. However, GC transcriptional activation can be mediated through interaction of the monomeric GC receptor with other DNA-bound TFs, such as *Ap1* and *Nfkb* [98]. In addition, a synergistic transcriptional activation has been reported between the GC receptor and members of the ETS TF family in different vertebrate species (summarized in Additional file 10). It should also be noted that the genes categorised in the present study as GC targets share several different TF binding sites with other network genes, and their expression might therefore have nothing to do with GC activity.

### Differences in network expression may affect matrix remodelling and ossification in the lower jaw and other orofacial elements

Common garden experiments focused on posthatching development have shown how head shape diverges among the Lake Thingvallavatn morphs. The contrasts are especially clear between the benthic and limnetic morphotypes and are reflected in functional elements, such as the relative length of the lower jaw [23]. This may be the result of a heterochronic effect whereby ossification of the dentary is initiated earlier in the benthic morphotypes (Eiriksson *et al.* and KHK unpublished data). At the molecular level, such divergences in the orofacial compartment can be directly associated with the network we have identified. During the morphogenetic process, the shape and size of craniofacial elements is rapidly changing, and these shape changes are likely to involve substantial matrix remodelling. Our results show that genes known to affect craniofacial morphogenesis that also play important roles in ECM deposition and remodelling display differential expression in the developing head of benthic and limnetic morphotypes. *Mmp2*, *Mmp9*, *Timp2*, *Ctsk* and *Sparc* all play roles in ECM remodelling [96, 100–103], and *Col1a1* and the proteoglycans *Lum*, *Ogn* and *Dcn* are components of the ECM [89, 104, 105]. Furthermore, many of these genes are directly and indirectly associated with the ossification process itself, for example, *Col1a1*, *Ctsk* and *Mmp2*, which play roles in intramembranous ossification [106].

Because of this tight association of the network with osteogenic and ECM sculpting processes, we postulate that the network could play an important role in the development of subtle differences in the trophic apparatus between morphotypes via spatial and/or temporal differences in ECM deposition and ossification. This is further supported by the observed overlap in expression patterns of selected genes from the network in the perichondrial region of the forming lower jaw (Figure 5), where ossification takes place. Thus, the differences in expression levels of the network genes might result in differences in the timing and level of ossification between the morphs, as well as other effects on cell shape, cellular arrangement and migration via matrix remodelling. Such variation may play a key role in speciation via trophic divergence. For example, transcriptional heterochrony causing a delay in ossification of the lower jaw has been associated with benthic to pelagic evolution in Antarctic notothenioid fish [26]. Similar suggestions have been voiced concerning trophic divergence in cichlids [8].

The overlapping expression of the proposed network genes in developing craniofacial elements in different morphotypes both supports the network model and indicates that differences in activity, rather than spatial expression patterns, underlie the observed differences in expression levels. This does not, however, exclude the possibility that differences in expression patterns of other genes may be of importance for shaping differences between morphotypes. Interestingly, the expression pattern of *Timp2* differed between the AC morph and the Lake Thingvallavatn morphs and showed visible differences in expression levels between the limnetic (PL) and benthic (SB and LB) morphs. *Timp2* has a highly conserved expressional correlation with the other network genes (see Figure 2) and was also the gene showing the strongest differences between benthic and limnetic morphs in the qPCR analysis (Additional file 2). It is conceivable that some members of a gene coexpression network are more sensitive than others to variation in the expression of regulatory genes, thus showing loss of expression in tissues where the expression of other members is still retained. Depending on chromatin state and the expression of other regulatory factors, this sensitivity may vary between tissues. This might explain how differences in expression levels could relate to morphological differences.

### Transcriptional regulators of the expression network

Having identified a network of differentially expressed genes with a potential role in crucial events which may underlie some of the differences between Arctic charr morphotypes, we were interested in discovering how this network might be differentially regulated between the morphs. A search for conserved motifs in three distant teleost fish species revealed potential binding sites for nine known TFs in the majority of the coexpressed genes (Table 1). Among these TFs, we found *Ap1* (*Jun*), ETS family, *Nfkb* and *Smad3/4* particularly interesting because of their characterized genetic interaction with genes of the network in other vertebrate species (summarized in Additional file 11). *Ap1* (activating protein 1) TFs are heterodimers of *Jun*, *Fos* or *Atf* that bind to a common DNA site [107]. Different *Ap1* factors can regulate different target genes in distinct biological processes, including normal development, depending on the relative abundance of *Ap1* subunits [107]. In addition, SMAD proteins are transcriptional modulators activated by transforming growth factor β (*Tgfβ*) that play a crucial role in regulating the specification of cranial neural crest cells [108]. Interestingly, interdependent cooperation and synergistic transactivation between *Ap1*, ETS family, *Nfkb* and SMADs have been reported by analysis of several responsive gene promoters in other vertebrates (Additional file 10). Two ETS family members (*Ets1* and *Ets2*) had already been selected for the present study as examples of less specific upstream effectors of GC transcriptional activity and because *Ets2* overexpression in mice has been reported to cause craniofacial abnormalities [109]. The fact that *Ets2* fits completely to the network described here is therefore of great interest. *Ets2* expression is higher in developing heads of benthic morphs and shows both positive correlation and spatiotemporal overlap with other network genes, which suggests a functional relationship. However, more detailed analysis is needed to evaluate whether *Ets2* (or some of the other predicted TFs) holds a potential master regulatory status in the coexpression network and morph-related differences and whether genetic separation in those or other regulators/binding sites have played a role in morph formation.

### Evolution of gene expression networks affecting craniofacial structures in teleost fish

Gene expression studies can provide novel and valuable insights into the molecular circuitry involved in the fine-tuning of variable morphology. Nevertheless, they can only imply, not directly identify, the triggers of the molecular cascades underlying variation and divergence. In general, causative genetic or environmental differences must underlie observed differences such as those seen in the tropic apparatus of Arctic charr morphs. The identification of causal genetic changes is not trivial [110]. Such changes can, for instance, affect the regulatory regions of the genes under study, regulatory regions of other genes and coding regions of genes or noncoding RNA genes. We recently profiled microRNA (miRNA) expression at four developmental stages in the AC and SB morphs and found significant expression differences in 53 previously described and 19 novel miRNA genes [111]. We are currently examining possible links between genes of the expression network described here and differentially expressed miRNAs. Furthermore, detailed analysis of coding and noncoding regions of the different charr populations is needed to identify potentially causal genetic variation. Recently, Attanasio and colleagues [112] demonstrated how distant acting enhancers act together to generate a complex pattern of gene expression in the craniofacial compartment during mouse development. Variations in regulatory regions were experimentally shown to cause changes in craniofacial morphology and were postulated to contribute to the diversity of craniofacial shape, not only in mice but also in humans [112]. These findings also support the common notion that changes in noncoding regulatory regions, which translate to differences in developmental gene expression, are at the heart of shaping morphological differences within and between species.

The upstream regulators of the gene network shown here to be differentially expressed in charr morphotypes are obviously of focal interest, the causalities of later developmental events are also of importance when studying morph-related differences. Using a common garden setup, Parsons *et al.* [24, 113] studied how different ‘food environments’ could affect shape variation within and among juveniles of Arctic charr morphs and morphotypes. In general, they found that the environment could have a considerable effect on morphology, indicating phenotypic plasticity at work. However, their results also strongly suggest that the more derived morphs (such as the benthic morphs) were less responsive to differences in the food environment, potentially indicating stronger canalization of developmental trajectories. We hypothesize that the genetic and developmental roots of such canalization, which appears to be further advanced in diverging systems such as the Lake Thingvallavatn morphs [113], could reside in differential deployment or tuning of networks of coregulated genes such as the ones described here.

## Conclusion

Our present study addresses the molecular mechanisms underlying the craniofacial divergence in the highly polymorphic Arctic charr. We have identified a network of coexpressed genes which are differentially expressed in benthic and limnetic morphotypes during developmental stages, spanning the emergence of craniofacial skeletal elements to hatching. Members of the gene network share biological functions mainly involved in ECM organization and skeletal formation. Moreover, coexpression connection between members of the network is conserved across distant vertebrate species, which suggests a crucial role of the genes in different developmental and physiological processes. The results of this study also predict a set of potential upstream TFs regulating the network. In particular, *Ets2*, a differentially expressed member of the ETS TF family shows a strong expression correlation and overlapping expression pattern in craniofacial structures with network genes. Identification of genetic differences, such as in *Ets2* or other regulators, influencing this conserved expression network can cast light on the agents used by evolution to shape trophic apparatus diversity in salmonids and possibly other fishes.

## Electronic supplementary material

Additional file 1: **Arctic charr–specific primers for cDNA cloning and qPCR.** (XLSX 13 KB)

Additional file 2: **Relative expression of candidate genes in the developing head of four Arctic charr morphs.** Relative expression of 12 candidate genes (with strong coexpression relationship in vertebrates) in the developing head of AC, PL, SB and LB at seven developmental stages. Gene expression was measured by qPCR, and expression levels were normalized with respect to the geometric means of two craniofacial reference genes (*Actb* and *If5a1*). The relative expression level for each gene is depicted by setting a replicate of the AC morph at stage 1 to an arbitrary unit of 1. Error bars represent standard deviation calculated from two biological replicates. The letters A, L, P and S above the bars indicate significantly higher expression than for AC, LB, PL and SB, respectively (*P* <0.05 calculated by Tukey’s HSD *post hoc* tests. (TIFF 499 KB)

Additional file 3: **Ranking of genes coexpressed with both** ***Mmp2*** **and** ***Sparc*** **,** **Gene Ontology (GO) and TF enrichment analyses.** Genes coexpressed with both *Mmp2* and *Sparc* in humans, mice and zebrafish were retrieved using COXPRESdb. The genes were filtered by setting the mutual rank (MR) to the top-ranked 500 and the reliability score of 3. In the list of *Mmp2* and *Sparc* coexpressed genes, the column titled ‘Transcriptome Presence’ indicates that an orthologous gene was present in Arctic charr transcriptome profiles. ‘Differential Expression’ indicates differences in expression levels between the AC and SB morphs in the same data. ‘Craniofacial Expression’ indicates craniofacial expression in zebrafish. The last two columns were used to select genes for further analysis in Arctic charr. The top-ranked (MR scores) of genes coexpressed with both *Mmp2* and *Sparc* for humans and mice were used as input for GO enrichment analysis using DAVID v6.7. GO terms with direct link to skeletal formation are highlighted in blue. Similar input lists of genes were used for transcription factor enrichment analysis in humans and mice through WebGestalt v2. Enriched TFs that were later predicted to be the potential regulators of the network are highlighted in orange. (XLSX 163 KB)

Additional file 4: **Analyses of candidate gene expression using two-way ANOVA for effects of morph and time.** Results show significant effects of morph, time and morph × time interaction on the expression of genes coexpressed with both *Mmp2* and *Sparc*, as well as selected GC downstream genes and upstream effectors. (XLSX 15 KB)

Additional file 5: **Analyses of candidate gene expression using two-way ANOVA for effects of morphotypes and time.** Results show significant effects of benthic and limnetic morphotypes, time and, to lesser extent, morphotype × time interaction on the expression of genes coexpressed with *Mmp2* and *Sparc*, as well as selected GC downstream genes and upstream effectors. (XLSX 15 KB)

Additional file 6: **Relative expression of eight downstream transcriptional targets of GC signalling.** Relative expression of candidate genes in the developing head of AC, PL, SB and LB at seven developmental stages, as measured by qPCR. Gene expression levels were normalized with respect to the geometric means of two craniofacial reference genes (*Actb* and *If5a1*). The relative expression level for each gene is depicted by setting a replicate of the AC morph at stage 1 to an arbitrary unit of 1. Error bars represent standard deviation calculated from two biological replicates. The letters A, L, P and S above the bars indicate significantly higher expression than in AC, LB, PL and SB, respectively (*P* <0.05) as calculated by Tukey’s HSD *post hoc* tests. (TIFF 358 KB)

Additional file 7: **Relative expression of eight upstream effectors of GC signalling.** Relative expression of candidate genes in the developing head of AC, PL, SB and LB at seven developmental stages, as measured by qPCR. Gene expression levels were normalized with respect to the geometric means of two craniofacial reference genes (*Actb* and *If5a1*). The relative expression level for each gene is depicted by setting a replicate of the AC morph at stage 1 to an arbitrary unit of 1. Error bars represent standard deviation calculated from two biological replicates. The letters A, L, P and S above the bars indicate significantly higher expression than in AC, LB, PL and SB, respectively (*P* <0.05) as calculated by Tukey’s HSD *post hoc* tests. (TIFF 339 KB)

Additional file 8: **Motif sequence enrichment analysis of candidate genes.** Two lists of motif sequences identified by SCOPE and transcription factor binding sites predicted by BioProspector in conserved noncoding regions of 17 coexpressed genes in three and four teleost fish species, respectively. The motifs identified by BioProspector were run against known PWMs in the TRANSFAC database using the STAMP tool. (XLSX 36 KB)

Additional file 9: **Craniofacial expression pattern of selected members of the coexpression network.** *In situ* hybridization revealing the anterior and ventral craniofacial expression pattern of *Ctsk*, *Mmp2*, *Ogn*, *Sfrp1*, *Sparc* and *Ets2* at stage 1 **(A)** and stage 7 **(B)**, ventral and lateral views showing the overlapping expression of the genes in the facial area anterior to and surrounding the mouth, as well as in the pharyngeal arches. *Sparc* displays ubiquitous and relatively less specific expression pattern during head development, and the expression of *Sfrp1* is hardly detectable at the last time point. Dashed red boxes emphasize on important expression patterns. Scale bar = 1 mM. (TIFF 6 MB)

Additional file 10: **A summary of known interactions between the predicted transcription factors in different vertebrate species.** (PDF 448 KB)

Additional file 11: **Regulatory interactions between predicted transcription factors and candidate genes in different vertebrate species.** (PDF 476 KB)

## Abbreviations

AC: Aquaculture fish stock
Cq: Cycle quantification
ECM: Extracellular matrix
GC: Glucocorticoid
LB: Large benthivorous charr
PI: Piscivorous charr
PL: Planktivorous charr
PWM: Motifs position weight matrix
RT-qPCR: reverse transcription quantitative polymerase chain reaction
RQ: Relative expression quantity
SB: Small benthivorous charr
TF: Transcription factor
τ_s_: Tau-somite relative age.
