# Supplementary figures and images for: Transcriptional dynamics of a conserved gene expression network associated with craniofacial divergence in Arctic charr
Source: EvoDevo. 2014 Nov 3;5:40. doi: 10.1186/2041-9139-5-40 (PMC4240837; doi:10.1186/2041-9139-5-40)

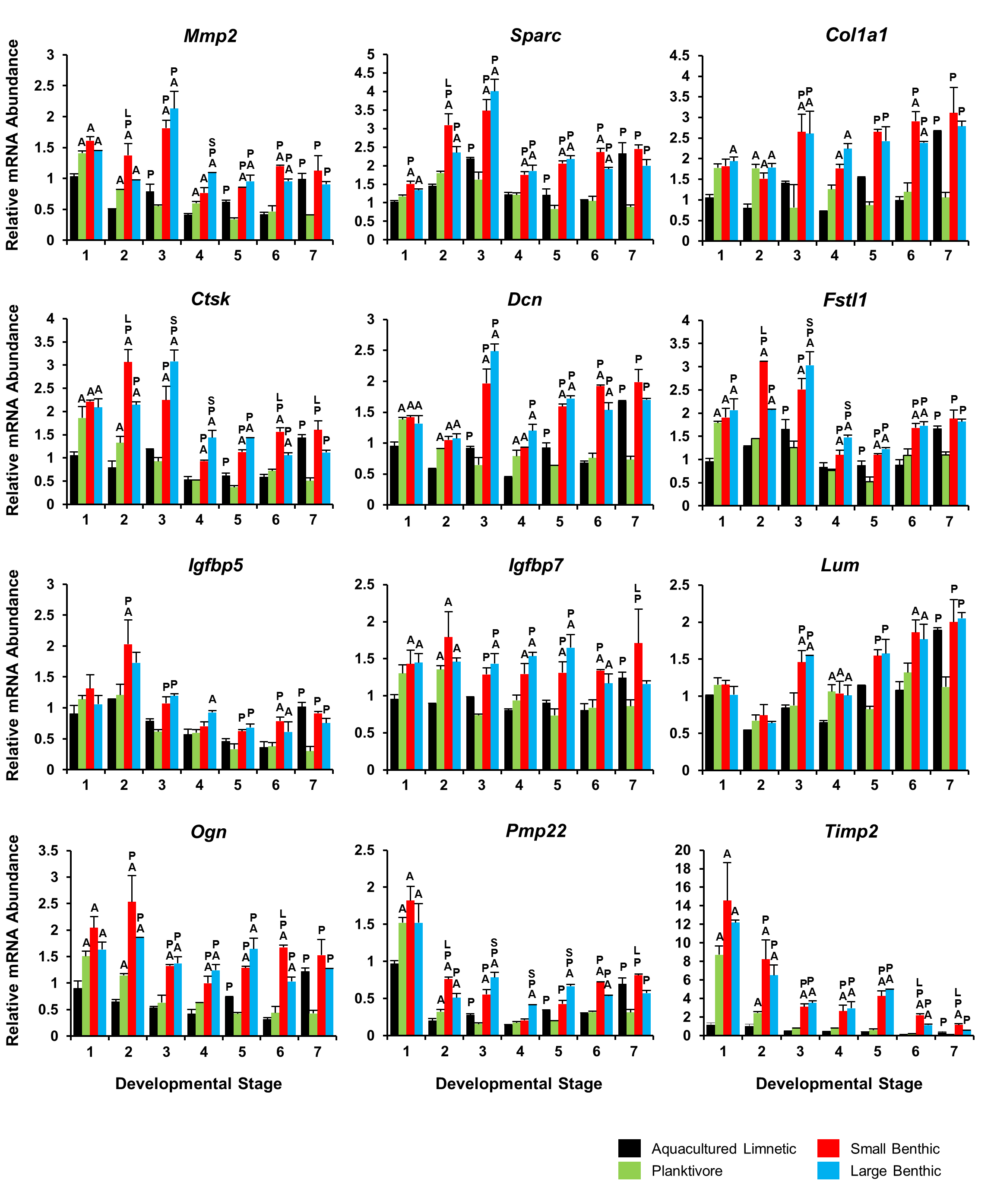

Supplement: Supplementary file 2 — Additional file 2: Relative expression of candidate genes in the developing head of four Arctic charr morphs. Relative expression of 12 candidate genes (with strong coexpression relationship in vertebrates) in the developing head of AC, PL, SB and LB at seven developmental stages. Gene expression was measured by qPCR, and expression levels were normalized with respect to the geometric means of two craniofacial reference genes (Actb and If5a1). The relative expression level for each gene is depicted by setting a replicate of the AC morph at stage 1 to an arbitrary unit of 1. Error bars represent standard deviation calculated from two biological replicates. The letters A, L, P and S above the bars indicate significantly higher expression than for AC, LB, PL and SB, respectively (P <0.05 calculated by Tukey’s HSD post hoc tests. (TIFF 499 KB) [file 13227_2014_122_MOESM2_ESM.tiff]

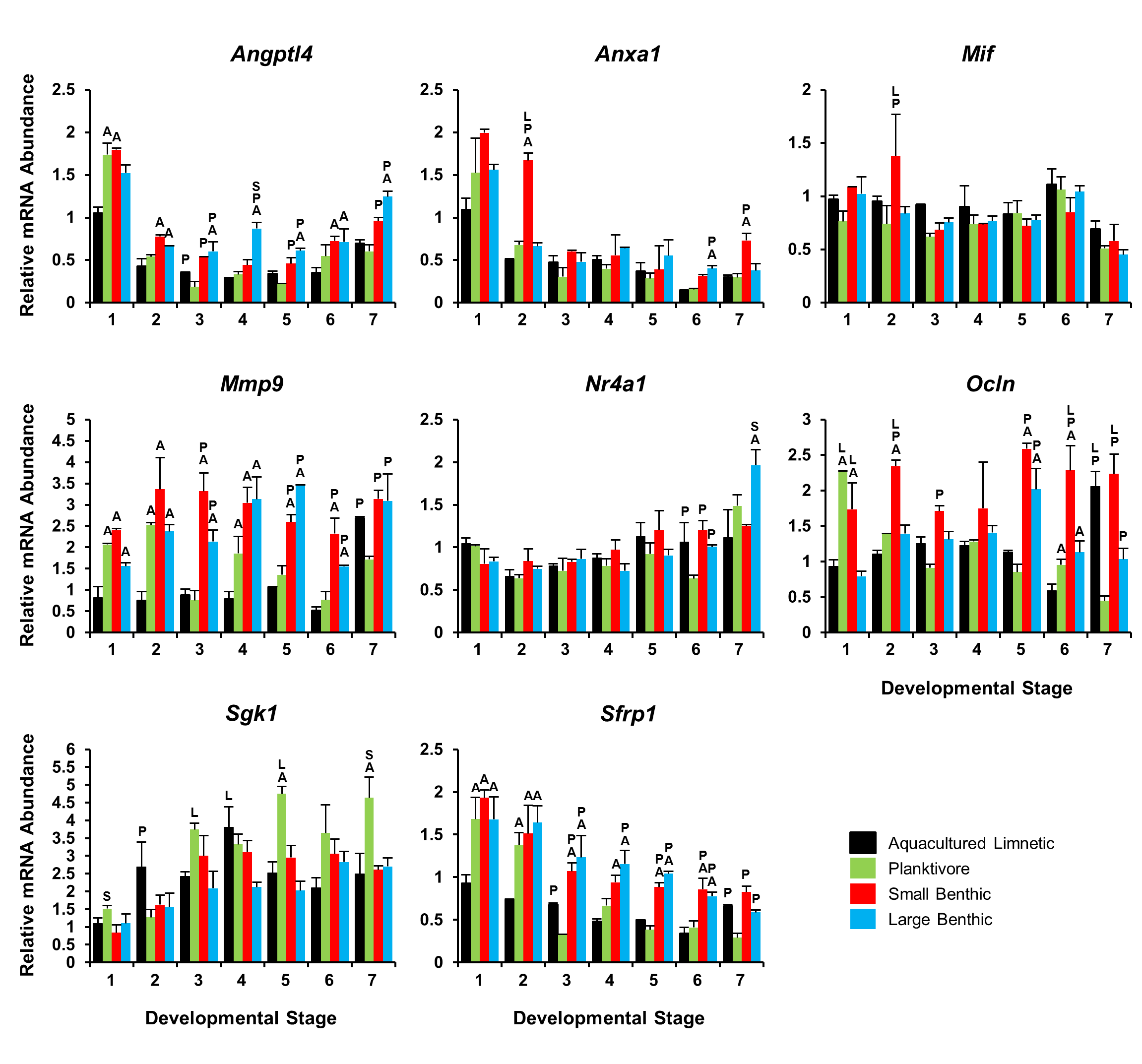

Supplement: Supplementary file 6 — Additional file 6: Relative expression of eight downstream transcriptional targets of GC signalling. Relative expression of candidate genes in the developing head of AC, PL, SB and LB at seven developmental stages, as measured by qPCR. Gene expression levels were normalized with respect to the geometric means of two craniofacial reference genes (Actb and If5a1). The relative expression level for each gene is depicted by setting a replicate of the AC morph at stage 1 to an arbitrary unit of 1. Error bars represent standard deviation calculated from two biological replicates. The letters A, L, P and S above the bars indicate significantly higher expression than in AC, LB, PL and SB, respectively (P <0.05) as calculated by Tukey’s HSD post hoc tests. (TIFF 358 KB) [file 13227_2014_122_MOESM6_ESM.tiff]

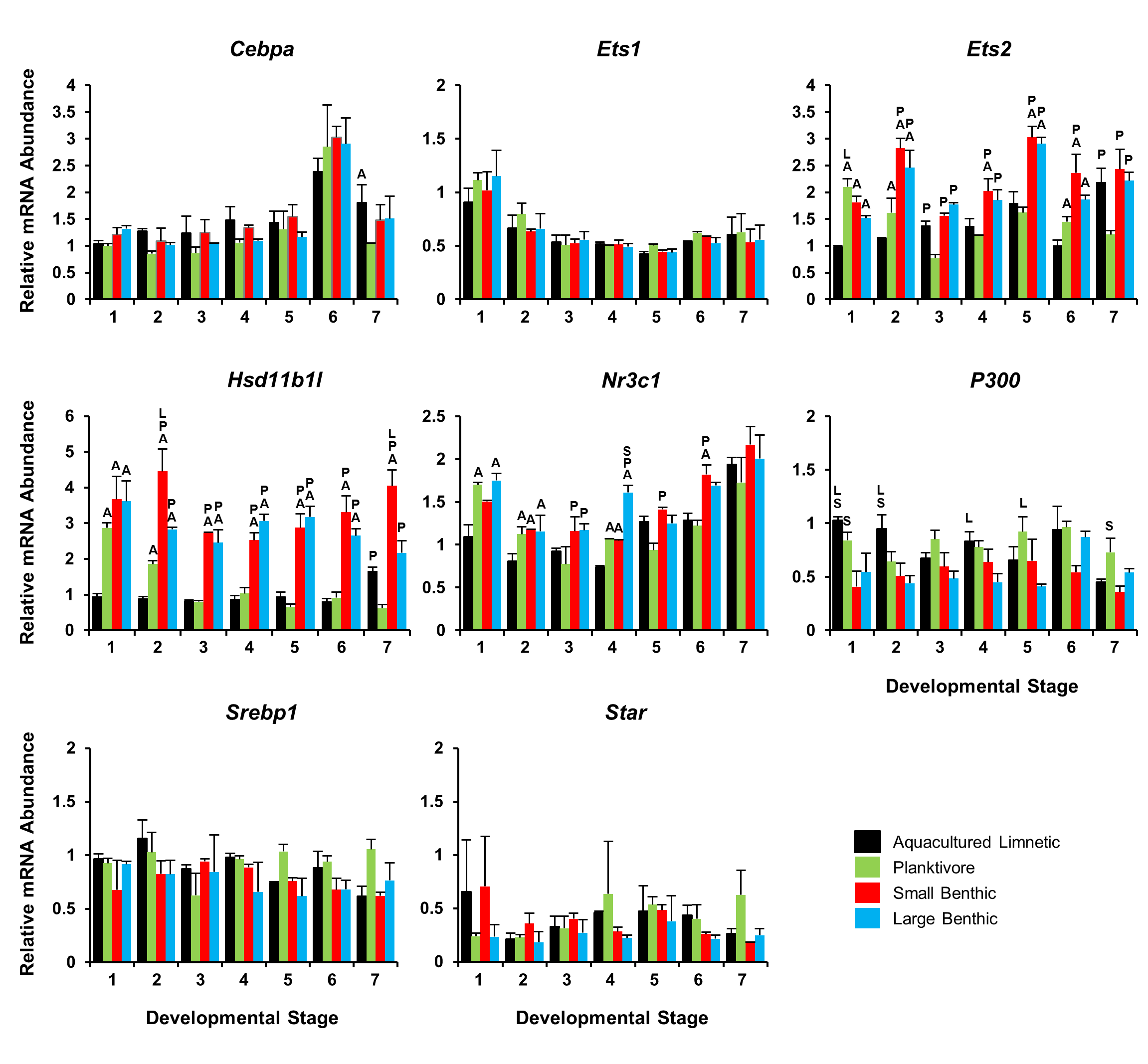

Supplement: Supplementary file 7 — Additional file 7: Relative expression of eight upstream effectors of GC signalling. Relative expression of candidate genes in the developing head of AC, PL, SB and LB at seven developmental stages, as measured by qPCR. Gene expression levels were normalized with respect to the geometric means of two craniofacial reference genes (Actb and If5a1). The relative expression level for each gene is depicted by setting a replicate of the AC morph at stage 1 to an arbitrary unit of 1. Error bars represent standard deviation calculated from two biological replicates. The letters A, L, P and S above the bars indicate significantly higher expression than in AC, LB, PL and SB, respectively (P <0.05) as calculated by Tukey’s HSD post hoc tests. (TIFF 339 KB) [file 13227_2014_122_MOESM7_ESM.tiff]

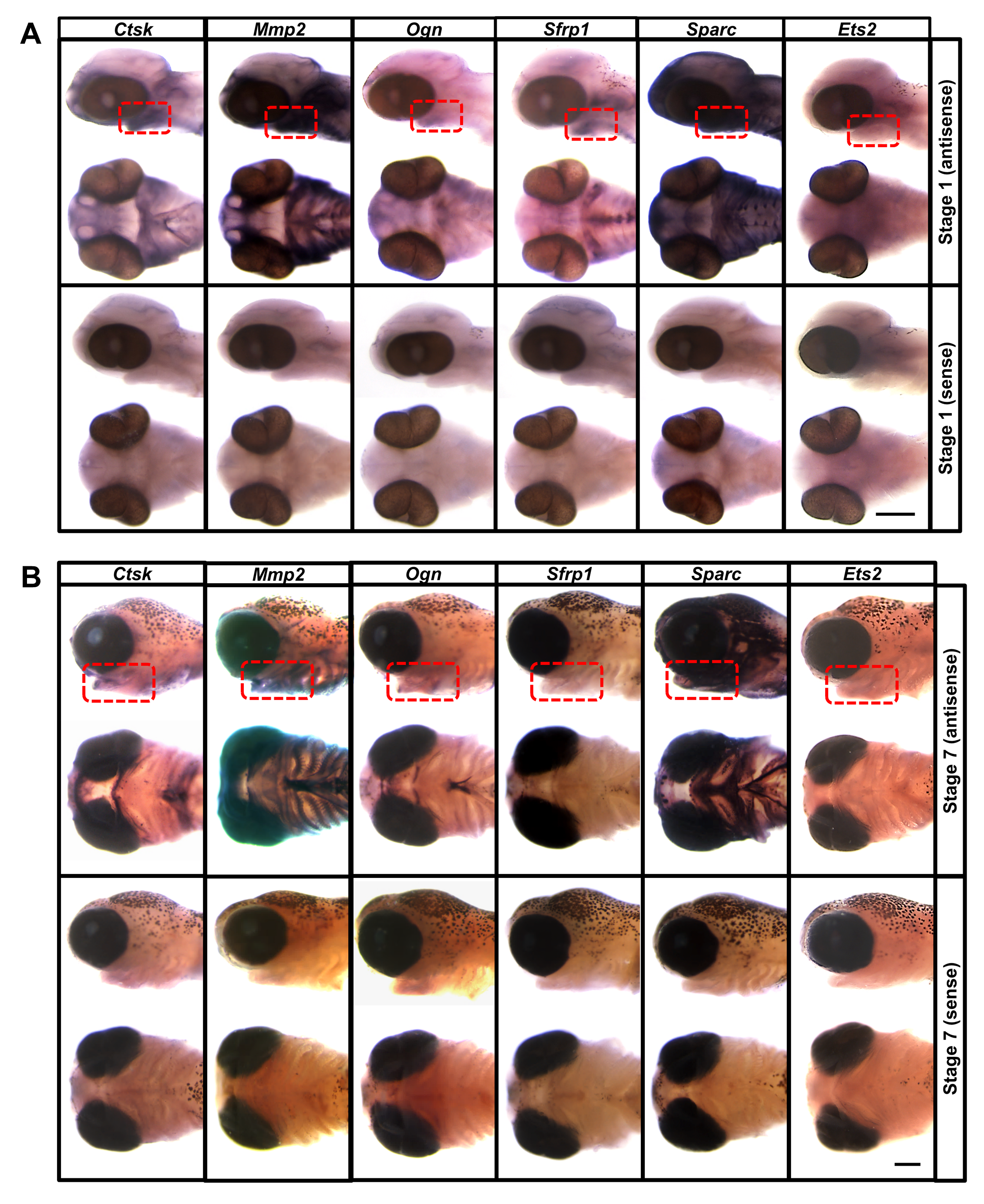

Supplement: Supplementary file 9 — Additional file 9: Craniofacial expression pattern of selected members of the coexpression network. In situ hybridization revealing the anterior and ventral craniofacial expression pattern of Ctsk, Mmp2, Ogn, Sfrp1, Sparc and Ets2 at stage 1 (A) and stage 7 (B), ventral and lateral views showing the overlapping expression of the genes in the facial area anterior to and surrounding the mouth, as well as in the pharyngeal arches. Sparc displays ubiquitous and relatively less specific expression pattern during head development, and the expression of Sfrp1 is hardly detectable at the last time point. Dashed red boxes emphasize on important expression patterns. Scale bar = 1 mM. (TIFF 6 MB) [file 13227_2014_122_MOESM9_ESM.tiff]
