# Supplementary material for: Transcriptional dynamics of a conserved gene expression network associated with craniofacial divergence in Arctic charr
Source: EvoDevo. 2014 Nov 3;5:40. doi: 10.1186/2041-9139-5-40 (PMC4240837; doi:10.1186/2041-9139-5-40)
Supplement: Supplementary file 10 — Additional file 10: A summary of known interactions between the predicted transcription factors in different vertebrate species. (PDF 448 KB) [file 13227_2014_122_MOESM10_ESM.pdf]

Additional file 10

A summary of known interactions between the predicted transcription factors in different vertebrate species

TFs	Type of Interaction	References
*Ap1/*ETS	Interdependent cooperation by adjacent binding sites	[1–5]
Ap1/ETS	Synergistic transcriptional activation	[6]
Ap1/ETS/*Nfkb*	Synergistic transcriptional activation	[7]
Ap1/*Nfkb*	Synergistic transcriptional activation	[8, 9]
Ap1/*Smad3-4*	Cooperative transcriptional activation	[10]
Ets1/Ets2	Transactivation through common binding site	[11–15]
ETS/GR (*Nr3c1*)	Synergistic transcriptional activation	[16–18]

References

1. Wasylyk B, Wasylyk C, Flores P, Begue A, Leprince D, Stehelin D: **The c-ets proto-oncogenes encode transcription factors that cooperate with c-Fos and c-Jun for transcriptional activation.** *Nature* 1990, **346**:191–3.

2. Bruder JT, Heidecker G, Rapp UR: **Serum-, TPA-, and Ras-induced expression from Ap-1/Ets-driven promoters requires Raf-1 kinase.** *Genes Dev* 1992, **6**:545–556.

3. Bergelson S, Daniel V: **Cooperative Interaction Between Ets and AP-1 Transcription Factors Regulates Induction of Glutathione S-Transferase Ya Gene Expression**. *Biochem Biophys Res Commun* 1994, **200**:290–297.

4. Cirillo G, Casalino L, Vallone D, Cesare D De, Verde P, Caracciolo A, Cesare DDE: **Role of Distinct Mitogen-Activated Protein Kinase Pathways and Cooperation between Human Urokinase-Type Plasminogen Activator Gene Induction by Interleukin-1 and Tetradecanoyl Phorbol Acetate Role of Distinct Mitogen-Activated Protein Kinase Pathways and**. *Mol Cell Biol* 1999, **19**:6240–6252.

5. Kim S, Denny CT, Wisdom R: **Cooperative DNA Binding with AP-1 Proteins Is Required for Transformation by EWS-Ets Fusion Proteins**. *Mol Cell Biol* 2006, **26**:2467–2478.

6. Logan SK, Garabedian MJ, Campbell CE, Werb Z: **Synergistic Transcriptional Activation of the Tissue Inhibitor of Metalloproteinases-1 Promoter via Functional Interaction of AP-1 and Ets-1 Transcription Factors**. *J Biol Chem* 1996, **271**:774–782.

7. Thomas RS, Tymms MJ, McKinlay LH, Shannon MF, Seth A, Kola I: **ETS1, NFkappaB and AP1 synergistically transactivate the human GM-CSF promoter.** *Oncogene* 1997, **14**:2845–55.

8. Adcock IM: **Transcription factors as activators of gene transcription: AP-1 and NF-kappa B.** *Monaldi Arch Chest Dis* 1997, **52**:178–86.

9. Mathas S, Hinz M, Anagnostopoulos I, Krappmann D, Lietz A, Jundt F, Bommert K, Mechta-Grigoriou F, Stein H, Dörken B, Scheidereit C: **Aberrantly expressed c-Jun and JunB are a hallmark of Hodgkin lymphoma cells, stimulate proliferation and synergize with NF-kappa B.** *EMBO J* 2002, **21**:4104–13.

10. Zhang Y, Feng XH, Derynck R: **Smad3 and Smad4 cooperate with c-Jun/c-Fos to mediate TGF-beta-induced transcription.** *Nature* 1998, **394**:909–13.

11. Harris T a, Yamakuchi M, Kondo M, Oettgen P, Lowenstein CJ: **Ets-1 and Ets-2 regulate the expression of microRNA-126 in endothelial cells.** *Arterioscler Thromb Vasc Biol* 2010, **30**:1990–7.

12. Petrovic N, Bhagwat S V, Ratzan WJ, Ostrowski MC, Shapiro LH: **CD13/APN transcription is induced by RAS/MAPK-mediated phosphorylation of Ets-2 in activated endothelial cells.** *J Biol Chem* 2003, **278**:49358–68.

13. Hasegawa Y, Abe M, Yamazaki T, Niizeki O, Shiiba K, Sasaki I, Sato Y: **Transcriptional regulation of human angiopoietin-2 by transcription factor Ets-1.** *Biochem Biophys Res Commun* 2004, **316**:52–8.

14. Wakiya K, Begue A, Stehelin D and, Shibuya M: **A cAMP Response Element and an Ets Motif Are Involved in the Transcriptional Regulation of flt-1 Tyrosine Kinase (Vascular Endothelial Growth Factor Receptor 1) Gene**. *J Biol Chem* 1996, **271**:30823–30828.

15. Wasylyk C, Gutman A, Nicholson R, Wasylyk B: **The c-Ets oncoprotein activates the stromelysin promoter through the same elements as several non-nuclear oncoproteins.** *EMBO J* 1991, **10**:1127–34.

16. Espinás ML, Roux J, Ghysdael J, Pictet R, Grange T: **Participation of Ets transcription factors in the glucocorticoid response of the rat tyrosine aminotransferase gene.** *Mol Cell Biol* 1994, **14**:4116–25.

17. Mullick J, Anandatheerthavarada HK, Amuthan G, Bhagwat S V, Biswas G, Camasamudram V, Bhat NK, Reddy SE, Rao V, Avadhani NG: **Physical interaction and functional synergy between glucocorticoid receptor and Ets2 proteins for transcription activation of the rat cytochrome P-450c27 promoter.** *J Biol Chem* 2001, **276**:18007–17.

18. Heuck-Knubel K, Proszkowiec-Weglarz M, Narayana J, Ellestad LE, Prakobsaeng N, Porter TE: **Identification of cis elements necessary for glucocorticoid induction of growth hormone gene expression in chicken embryonic pituitary cells.** *Am J Physiol Regul Integr Comp Physiol* 2012, **302**:R606–19.
