# Supplementary material for: Transcriptional dynamics of a conserved gene expression network associated with craniofacial divergence in Arctic charr
Source: EvoDevo. 2014 Nov 3;5:40. doi: 10.1186/2041-9139-5-40 (PMC4240837; doi:10.1186/2041-9139-5-40)
Supplement: Supplementary file 11 — Additional file 11: Regulatory interactions between predicted transcription factors and candidate genes in different vertebrate species. (PDF 476 KB) [file 13227_2014_122_MOESM11_ESM.pdf]

Additional file 11

Regulatory interaction between predicted transcription factors and candidate genes in different vertebrate species

Gene	Species	TF	Binding Site	TF Action	References
Angptl4	Human	*Ap1*	Yes	Induction	[1]
	Human	ETS	Yes	Induction	[1]
	Human	SMAD	Yes	Induction	[1]
Ctsk	Human, Mouse	*Ap1*	Yes	?	[2, 3]
	Human, Mouse	ETS	Yes	?	[2, 3]
	Mouse	*Nfkb*	Yes	?	[3]
	Mouse	*Smad4*	?	Repression	[4]
Dcn	Human	*Ap1*	Yes	Repression	[5, 6]
	Human	ETS	Yes	Induction	[7]
	Human	*Nfkb*	Yes	?	[6]
Igfbp5	Human, Rat	*Ap1*	Yes	?	[8, 9]
	Human	ETS	Yes	Induction	[7]
Lum	Human, Mouse	ETS	Yes	Induction	[7, 10]
	Mouse	*Smad3*	?	Induction	[11]

Mmp2	Human	*Ap1*	Yes	Induction	[12–14]
	Human	ETS	Yes	Induction	[13–15]
	Human, Mouse	*Smad3*	?	Induction	[16–18]
Mmp9	Human, Rat	*Ap1*	Yes	Induction	[13, 14, 19]
	Human	ETS	Yes	Induction	[13, 14, 20]
	Human, Rat	*Nfkb*	Yes	Repression/ Induction	[19, 21]
	Human	SMAD	Yes	Repression	[21]
Sfrp1	Human, Rat	*Ap1*	Yes	?	[22]
	Human, Rat	ETS	Yes	?	[22]
Sparc	Chicken, Human	*Ap1*	Yes	Repression/ Induction	[12, 23]
	Chicken, Human, Mouse, Cow	ETS	Yes	?	[23]
	Human	*Smad3*	?	Induction	[24]
Timp2	Human	*Ap1*	Yes	Induction	[14]
	Human	ETS	Yes	Induction	[14]

References

1. Kaddatz K, Adhikary T, Finkernagel F, Meissner W, Müller-Brüsselbach S, Müller R: **Transcriptional profiling identifies functional interactions of TGF β and PPAR β/δ signaling: synergistic induction of ANGPTL4 transcription.** *J Biol Chem* 2010, **285**:29469–79.

2. Rood J a, Van Horn S, Drake FH, Gowen M, Debouck C: **Genomic organization and chromosome localization of the human cathepsin K gene (CTSK).** *Genomics* 1997, **41**:169–76.

3. Li Y-P, and, Chen W: **Characterization of Mouse Cathepsin K Gene , the Gene**. *J BONE Miner Res* 1999, **14**:487–499.

4. Tan X, Weng T, Zhang J, Wang J, Li W, Wan H, Lan Y, Cheng X, Hou N, Liu H, Ding J, Lin F, Yang R, Gao X, Chen D, Yang X: **Smad4 is required for maintaining normal murine postnatal bone homeostasis.** *J Cell Sci* 2007, **120**(Pt 13):2162–70.

5. Mauviel, A., Korang, K., Santra, M., Tewari, D., Uitto, J., & Iozzo R V.: **Identification of a Bimodal Regulatory Element Encompassing a Canonical AP-1 Binding Site in the Proximal Promoter Region of the Human Decorin Gene**. *J Biol Chem* 1996, **271**:24824–24829.

6. Santra M, Danielson KG, Renato V: **Structural and Functional Characterization of the Human Decorin Gene Promoter**. *J Biol Chem* 1994, **269**:579–587.

7. Hahne JC, Okuducu AF, Fuchs T, Florin A, Wernert N: **Identification of ETS-1 target genes in human fibroblasts.** *Int J Oncol* 2011, **38**:1645–52.

8. Zhu X, Ling N, Shimasaki S: **Cloning of the rat insulin- like growth factor binding protein-5 gene and DNA sequence analysis of its promoter region.** *Biochem Biophys Res Commun* 1993, **190**:1045–52.

9. Niu J, Huang Y-J, Wei S, Liu Z, Wang L-E, Chang S, Chamberlain RM, El-Naggar AK, Sturgis EM, Wei Q: **Association between a functional polymorphism (-1195T>C) in the IGFBP5 promoter and head and neck cancer risk.** *Head Neck* 2011, **33**:650–60.

10. Li V, Raouf A, Kitching R, Seth A: **Ets2 transcription factor inhibits mineralization and affects target gene expression during osteoblast maturation.** *In Vivo* 2004, **18**:517–24.

11. Saika S, Kono-Saika S, Tanaka T, Yamanaka O, Ohnishi Y, Sato M, Muragaki Y, Ooshima A, Yoo J, Flanders KC, Roberts AB: **Smad3 is required for dedifferentiation of retinal pigment epithelium following retinal detachment in mice.** *Lab Invest* 2004, **84**:1245–58.

12. Hayakawa J, Mittal S, Wang Y, Korkmaz KS, Adamson E, English C, Ohmichi M, Omichi M, McClelland M, Mercola D: **Identification of promoters bound by c-Jun/ATF2 during rapid large-scale gene activation following genotoxic stress.** *Mol Cell* 2004, **16**:521–35.

13. Crawford HC, Matrisian LM: **Mechanisms controlling the transcription of matrix metalloproteinase genes in normal and neoplastic cells.** *Enzyme Protein* 1996, **49**:20–37.

14. Borden P, Heller RA: **Transcriptional control of matrix metalloproteinases and the tissue inhibitors of matrix metalloproteinases.** *Crit Rev Eukaryot Gene Expr* 1997, **7**:159–78.

15. Staun-Ram E, Goldman S, Shalev E: **Ets-2 and p53 mediate cAMP-induced MMP-2 expression, activity and trophoblast invasion.** *Reprod Biol Endocrinol* 2009, **7**:135.

16. Bujak M, Ren G, Kweon HJ, Dobaczewski M, Reddy A, Taffet G, Wang X-F, Frangogiannis NG: **Essential role of Smad3 in infarct healing and in the pathogenesis of cardiac remodeling.** *Circulation* 2007, **116**:2127–38.

17. Verrecchia F, Chu ML, Mauviel A: **Identification of novel TGF-beta /Smad gene targets in dermal fibroblasts using a combined cDNA microarray/promoter transactivation approach.** *J Biol Chem* 2001, **276**:17058–62.

18. Lee JW, Zoumalan R a, Valenzuela CD, Nguyen PD, Tutela JP, Roman BR, Warren SM, Saadeh PB: **Regulators and mediators of radiation-induced fibrosis: Gene expression profiles and a rationale for Smad3 inhibition.** *Otolaryngol Head Neck Surg* 2010, **143**:525–30.

19. Takahra T, Smart DE, Oakley F, Mann D a.: **Induction of myofibroblast MMP-9 transcription in three-dimensional collagen I gel cultures: regulation by NF-κB, AP-1 and Sp1**. *Int J Biochem Cell Biol* 2004, **36**:353–363.

20. Watabe T, Yoshida K, Shindoh M, Kaya M, Fujikawa K, Sato H, Seiki M, Ishii S, Fujinaga K: **The Ets-1 and Ets-2 transcription factors activate the promoters for invasion-associated urokinase and collagenase genes in response to epidermal growth factor**. *Int J cancer* 1998, **137**:128–137.

21. Ogawa K, Chen F, Kuang C, Chen Y: **Suppression of matrix metalloproteinase-9 transcription by transforming growth factor-beta is mediated by a nuclear factor-kappaB site.** *Biochem J* 2004, **381**(Pt 2):413–22.

22. Katoh Y, Katoh M: **Comparative genomics on SFRP1 orthologs.** *Int J Oncol* 2005, **27**:861–5.

23. Vial E, Perez S, Castellazzi M: **Transcriptional control of SPARC by v-Jun and other members of the AP1 family of transcription factors.** *Oncogene* 2000, **19**:5020–9.

24. Kang MH, Oh D-J, Kang J, Rhee DJ: **Regulation of SPARC by transforming growth factor β2 in human trabecular meshwork.** *Invest Ophthalmol Vis Sci* 2013, **54**:2523–32.
